# The effect of ankle‐foot orthoses on gait characteristics in people with Charcot‐Marie‐Tooth disease: A systematic review and meta‐analysis

**DOI:** 10.1002/jfa2.70003

**Published:** 2024-09-14

**Authors:** Andrew Kim, Mike Frecklington, Adam Philps, Sarah Stewart

**Affiliations:** ^1^ Department of Podiatry Auckland University of Technology Auckland New Zealand; ^2^ Masterton Foot Clinic Masterton New Zealand

**Keywords:** ankle‐foot orthoses, Charcot‐Marie‐Tooth disease, meta‐analysis, systematic review

## Abstract

**Introduction:**

Ankle‐foot orthoses (AFOs) are commonly prescribed for people with Charcot‐Marie‐Tooth disease (CMT) to improve gait efficiency and reduce the occurrence of tripping and falls. The aim of this study was to systematically review evidence on the effects of AFOs on gait kinematics and kinetics and postural stability/balance in people with CMT.

**Methods:**

Studies were identified from electronic databases and screened for inclusion online using Rayyan. Data from all eligible studies were extracted into a standardised Excel spreadsheet. Methodological quality was assessed using the Joanna Briggs Institute Critical Appraisal Checklists. Where available, continuous outcomes were pooled to estimate standardised mean differences in random‐effects meta‐analyses.

**Results:**

A total of 15 studies were included with variable methodological quality. Sample sizes ranged from 1 to 32 with significant variability in participant characteristics, AFO designs and testing procedures. Data from eight studies were available for meta‐analysis. Although AFOs impacted walking velocity, stride length, step length, cadence, ankle dorsiflexion, plantarflexion, knee and hip flexion and ankle plantarflexion and dorsiflexion moments, the effect sizes were small‐to‐moderate and non‐significant. There were insufficient data available for pooled analyses of outcomes related to postural stability/balance.

**Conclusion:**

Although AFOs positively affect a number of gait and balance parameters, the small participant numbers, variability in participant characteristics, AFO designs and testing procedures adopted by the available studies resulted in the absence of statistically significant effects when data were pooled. The results from this review also highlight the importance of device customisation based on the individual needs of people with CMT and their degree of gait impairment.

## 1 INTRODUCTION

Charcot‐Marie‐Tooth disease (CMT) is the most common group of inherited peripheral neuropathies. The clinical presentation of CMT is characterised by a slow and progressive neuropathy, with distal weakness and atrophy in the limbs being the most common initial presentation [1]. Sensory ataxia and decreased‐to‐absent deep tendon reflexes may also be present [2]. Foot deformities, including pes cavus and hammer toes, are seen in up to 74% of people with CMT, as well as progressive atrophy of intrinsic foot muscles [3]. Over time, these deformities can become fixed and cause laxity of the ankle joint, relative dorsiflexion of the talus, and eventual loss of mobility of the ankle joint [4, 5, 6].

Laboratory‐based gait analyses have identified several altered gait patterns in people with CMT [7]. Foot drop, resulting from a loss of sagittal plane ankle stability is the most prominent compensatory gait pattern seen in people with CMT, which results in a lack of heel contact during initial contact and a loss of propulsive power [7, 8]. An imbalance between weaker evertor muscles and stronger inverter muscles also contributes to abnormal frontal plane foot function, further impairing overall gait [9]. As a result, people with CMT fall frequently, experience difficulties with undertaking activities of daily living and report overall reduced quality of life [10, 11].

Ankle‐foot orthoses (AFOs) are commonly prescribed in people with CMT to improve gait efficiency, reduce energy costs and reduce the risk of tripping and falling by compensating for the lack of propulsion during stance [11, 12]. There are many AFO designs available which are prescribed according to the patients' individual needs [13] and their reported use among people with CMT ranges from 19% to 49% [3, 14, 15]. Only two reviews of the effect of AFOs in people with CMT have been published; however, they were narrative reviews and did not adopt a systematic approach or include pooled meta‐analyses [16, 17]. Therefore, the aim of the current study was to systematically review evidence on the biomechanical effects of AFOs in people with CMT, including their impact on gait kinematics, gait kinetics and postural stability/balance.

## 2 METHODS

### 2.1 Study design

The development and reporting of this study were guided by the preferred reporting items for systematic reviews and meta‐analyses (PRISMA) guidelines [18]. The Cochrane Handbook for Systematic Reviews of Interventions [19] was also used to guide the approach taken to study identification, data extraction, quality assessment and data analysis. This review has been registered with the International Prospective Register of Systematic Reviews (PROSPERO CRD42023451522).

### 2.2 Study eligibility

All forms of primary research designs were considered for inclusion, including randomised controlled trials (RCTs), quasi‐experimental studies, cross‐sectional comparison studies and case studies. Only studies involving participants with a physician diagnosis of CMT were included. There were no restrictions placed on recruitment setting, participant gender, age, duration of disease or disease severity. Participants with all CMT types were considered for inclusion. Studies evaluating any type of AFO were considered for inclusion, including both prefabricated and customised designs. Due to the lack of RCTs later identified in this review, acceptable comparators included shoes only, barefoot or other AFOs (rather than ‘no intervention, a placebo/sham intervention, advice to rest/usual care, or any other alternative intervention’ as reported in the PROSPERO protocol). There were no restrictions placed on the duration of the intervention. To be included, studies had to report outcomes of interest related to changes in gait kinematics, gait kinetics and/or postural stability/balance between the intervention and comparator group(s). There were no restrictions on the measurement tools used to assess these outcomes. Non‐English language studies were excluded. Conference abstracts were excluded due to incomplete reporting of methodology and results. Studies that reported data or secondary analyses of data presented in an already included study were excluded. Studies involving AFOs as part of a larger rehabilitation program (i.e., including physical exercise, surgery, etc.) were excluded due to the inability to determine the effect of these devices independent of other rehabilitation strategies.

### 2.3 Search strategy

The following electronic databases were searched from inception to December 2023: CENTRAL (via Wiley), MEDLINE (via Ovid), Allied and Complementary Medicine (AMED) (via Ovid), The Cumulative Index to Nursing and Allied Health Literature (CINAHL) Complete (via EBSCO), SportDiscus with Full Text (via EBSCO), Physiotherapy Evidence Database (PEDro), Scopus and Google Scholar. These included databases slightly deviate from the registered PROSPERO protocol as a result of preliminary searches and further refinement of the search strategy. This included removal of Embase and the addition of Google Scholar to balance efficiency and comprehensiveness [20, 21]. Search terms are presented in Supporting Information S1. The search was also supplemented by manual searching of reference lists of relevant review articles. All identified studies were exported into Rayyan software [22] and duplicates were removed automatically. Two researchers (AK, SS) then independently screened all identified studies against the above eligibility criteria based on the titles, keywords and abstracts. Subsequently, full‐text manuscripts were independently assessed for eligibility by the same two independent researchers (AK, SS). Any disagreements between researchers were resolved by discussion to achieve consensus.

### 2.4 Data extraction and management

Two researchers (AK, SS) extracted data into a standardised Microsoft Excel spreadsheet. Extracted data included study characteristics (first author surname, article title, publication year and research design), participant characteristics (number, age, gender, CMT subtype and inclusion/exclusion criteria), intervention characteristics (description of AFO, description of comparator and key protocol details), description of outcomes related to gait kinematics, gait kinetics and postural stability/balance and the results of these outcomes across interventions (mean and standard deviation or median and interquartile range). Any disagreements between the two researchers were resolved through consensus and discussion.

### 2.5 Quality assessment

Methodological quality of the included studies was assessed using the Joanna Briggs Institute (JBI) Critical Appraisal Checklists [23] due to the wide range of study designs represented across the included studies, including the JBI Critical Appraisal Tools for quasi‐experimental (non‐randomised studies) and case reports. Due to the absence of RCTs later identified in this review and the decision to use a single standardised tool across all included studies, this tool differs from those reported in the PROPSERO protocol (Cochrane Risk of Bias 2 tool for randomised trials [24] and Cochrane risk of bias in non‐randomised studies (ROBINS‐I) tool [25]). JBI checklists allow for rigorous appraisal of the presence of any bias in design, conduct and analysis of research studies. Two researchers (AK, MF) independently evaluated the quality of included studies, and any disagreements were resolved by discussion.

### 2.6 Meta‐analyses

Meta‐analyses were conducted to estimate the pooled standardised mean differences (SMDs) and their 95% confidence intervals (CIs) as a representation of effect size for differences in outcome measures between AFO and comparator (control) groups. Only studies which reported outcome results as mean and SD for both the intervention and comparator groups were considered for inclusion in the meta‐analyses. Data from case studies and data from studies reporting results in medians and ranges were therefore excluded from the meta‐analyses. For studies reporting results separately for right and left limbs, the average values from both limbs were calculated and used for analysis. Where outcomes were reported in different measurement units, values were converted to the same measurement unit if possible (e.g., for studies reporting stride length in mm, cm or m, all data were converted to the same measurement unit (cm) prior to analysis). For studies measuring gait outcomes at multiple self‐selected speeds (e.g., fast, comfortable and slow walking speeds), only data measured during comfortable walking speeds were included in the meta‐analyses. Comparator groups, regardless of whether they were barefoot or shoes only or a mixture of both were considered together under 'control' due to the reported absence of differences in gait parameters between barefoot and shoe conditions [26]. For studies comparing more than one AFO intervention with a comparator (and therefore contributing more than one effect size to the analysis), the means and standard deviations for all AFO interventions were pooled to avoid double‐counting and correlated effect sizes (as recommended by Cochrane [27]).

Random effects models were used for all analyses due to the high level of anticipated heterogeneity in the true effects as a result of the various AFO types, control conditions, and outcome measurement protocols used across the included studies [28]. Due to the continuous nature of outcome data, the restricted maximum likelihood estimator was used in all analyses to estimate the heterogeneity variance (the distribution of true effect sizes) (Tau^2^) [29]. Confidence intervals around the pooled SMDs were computed using Knapp–Hartung adjustments [30], which are recommended when few studies are available for pooling [31, 32]. As the sample sizes of the included studies were relatively small, the SMDs were corrected using Hedges' *g* to reduce overestimation of the true effect size due to the small sample bias [33]. Hedges' *g* SMDs were interpreted using the conventions by Cohen [33] where a small effect = 0.20, moderate effect = 0.50 and large effect = 0.80. Positive values were interpreted as an increase in the outcome measure in AFO conditions over the comparator. *I* ^2^ was used to quantify the effect of heterogeneity where a value of 0% indicated no observed heterogeneity, and larger values showed increasing heterogeneity [34]. All meta‐analyses were conducted using the *meta*, *dmetar*, and *metafor* packages in RStudio (version 2023.06.1 + 524).

## 3 RESULTS

### 3.1 Search results

**FIGURE 1:**
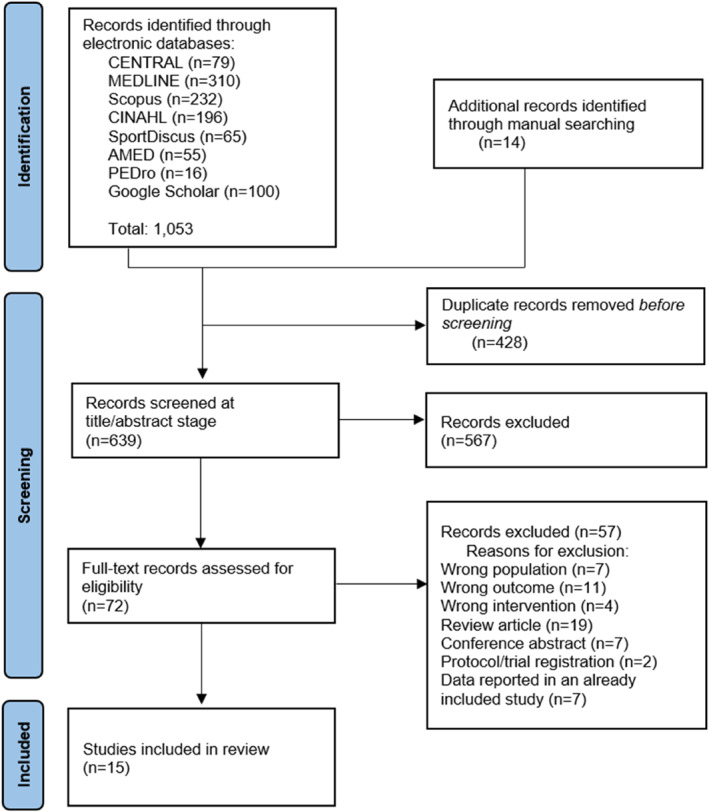
PRISMA flow chart.

A total of 1053 studies were identified across the searched databases (Figure 1). After removal of duplicates, 639 studies were screened based on title and abstract, of which 72 were included in the full‐text screening stage. Of these, 57 were excluded (reasons described in Figure 1), and a total of 15 studies were included in the review.

### 3.2 Study and participant characteristics

Study and participant characteristics are presented in Table 1. Ten studies were quasi‐experimental studies and five were case reports. Sample sizes ranged from 1 to 32, with a total of 116 participants with CMT across all included studies. Participants were predominantly male (*n* = 66, 56.9%) and mean ages ranged from 11 to 57 years. Outcomes measured across the included studies included gait kinematics (assessed in 12 studies), gait kinetics (*n* = 7 studies) and postural stability/balance (*n* = 4 studies). One study reported on all three outcomes, 11 studies reported on two and three studies reported on one.

**TABLE 1:** Study and participants' characteristics of included studies (*n* = 15 studies).

Study	Research design	Participant characteristics				Interventions and comparators	Outcomes measured
		Number	Age (years)	Gender	CMT subtype(s)		
Bean et al. 2001 [35]	Case study	1	51	1 male	NR	**Comparator**: Non‐customised posterior leaf spring AFO	Gait kinetics
						**Intervention 1**: Customised semi‐rigid AFO	
Borghi et al. 2023 [36]	Within‐subject comparison	3	Mean 14	1 female: 2 male	NR	**Comparator**: Barefoot	Gait kinematics; gait kinetics; postural stability/balance
						**Intervention 1**: Customised silicone AFO	
						**Intervention 2**: Customised botter AFO	
Burdett & Hassell 2004 [37]	Case study	1	40	1 male	NR	**Comparator**: Shoes only	Gait kinematics; gait kinetics
						**Intervention 1**: Customised solid ankle AFO	
						**Intervention 2**: Customised posterior leaf spring AFO	
						**Intervention 3**: Pre‐fabricated AFO	
Burke et al. 2021 [38]	Within‐subject comparison	9	Mean 52	6 female: 3 male	CMT1A (*n* = 6), CMT4C (*n* = 1), unknown (*n* = 2)	**Comparator**: Shoes only	Postural stability/balance
						**Intervention 1**: Dynamic carbon ground reaction AFO	
Del Bianco & Fatone 2008 [39]	Case study	1	49	1 male	CMT1X (*n* = 1)	**Comparator**: Shoes only	Gait kinematics; gait kinetics
						**Intervention 1:** Customised posterior leaf spring AFO	
						**Intervention 2**: Customised silicone AFO	
Dufek et al. 2014 [40]	Within‐subject comparison	8	Mean 55.7	3 female: 5 male	Not reported	**Comparator**: Shoes only	Gait kinematics; gait kinetics
						**Intervention 1**: Customised carbon‐fibre composite AFO	
Guillesbastre et al. 2011 [41]	Within‐subject randomised comparison	26	Mean 50.7	11 female: 15 male	Not reported	**Comparator**: Shoes with/without usual brace	Gait kinematics; postural stability/balance
						**Intervention 1**: Plastic AFO	
						**Intervention 2**: Elastic AFO	
Menotti et al. 2014 [42]	Within‐subject randomised comparison	7	Mean 37	4 female: 3 male	Type 1A	**Comparator**: Shoes only	Gait kinetics; gait kinematics
						**Intervention 1**: Anterior elastic AFO	
Ounpuu et al. 2021 [43]	Within‐subject comparison	15	Mean 12	6 female: 9 male	CMT1 (*n* = 5), CMT 2 (*n* = 6), CMT4E (*n* = 1), unknown (*n* = 3)	**Comparator**: Barefoot	Gait kinematics; gait kinetics
						**Intervention 1**: AFO	
Pereira et al. 2014 [44]	Within‐subject comparison	9	Mean 41	5 female: 4 male	Not reported	**Comparator**: Shoes only	Gait kinematics; postural stability/balance
						**Intervention 1**: AFO	
Phillips et al. 2012 [45]	Within‐subject randomised comparison	8	Mean 57	3 female: 5 male	CMT disease type 1 or 2.	**Comparator**: Barefoot or shoes only	Gait kinematics
						**Intervention 1**: Customised silicone AFO	
						**Intervention 2**: Customised polypropylene AFO	
						**Intervention 3**: Prefabricated AFO	
Ramdharry et al., 2012 [46]	Within‐subject comparison	14	Mean 38	5 female: 9 male	CMT1a (*n* = 11), CMTX (*n* = 5), CMT2 (*n* = 4), HSN1 (*n* = 2)	**Comparator**: Shoes only	Gait kinematics; gait kinetics
						**Intervention 1**: Prefabricated anterior elastic AFO	
						**Intervention 2**: Prefabricated push brace AFO	
						**Intervention 3**: Posterior leaf AFO	
Van der Wilk et al. 2018 [47]	Case study	1	59	1 male	Not reported	**Comparator**: Participant's existing AFO	Gait kinematics; gait kinetics
						**Intervention 1**: ADJUST AFO stiff	
						**Intervention 2**: ADJUST AFO stiff/flexible	
						**Intervention 3**: ADJUST AFO flexible	
						**Intervention 4**: ADJUST AFO flexible/stiff	
Vinci et al. 2010 [48]	Case study	1	50	1 male	CMT 2A2 (*n* = 1)	**Comparator**: Shoes only	Gait kinematics
						**Intervention 1**: Customised silicone AFO	
						**Intervention 2**: Codivilla	
						**Intervention 3**: Soft footdrop insert	
Wojciechowski et al. 2022 [49]	Within‐subject randomised comparison	12	Mean 11.2	6 female: 6 male	CMT1A (*n* = 5), CMT1E (*n* = 1), CMTX3 (*n* = 2), CMT2 (*n* = 1), CMT2D (*n* = 1), CMT4C (*n* = 2)	**Comparator**: Shoes only	Gait kinematics; gait kinetics
						**Intervention 1**: Traditional AFO	
						**Intervention 2**: 3D printed replica AFO	
						**Intervention 3**: 3D printed redesigned AFO	

Abbreviations: 3D, three dimensional; AFO, ankle foot orthoses; CMT, Charcot‐Marie‐Tooth; NR, not reported.

### 3.3 AFO characteristics

A range of comparator and AFO interventions were assessed across the included studies (Table 1). The majority of studies compared AFOs to shoes only (*n* = 10). Other comparator conditions included barefoot (*n* = 2), barefoot or shoes (*n* = 1) and other AFOs (*n* = 2). Most studies investigated a single AFO intervention (*n* = 6), followed by three AFO interventions (*n* = 5), two AFO interventions (*n* = 3) and four AFO interventions (*n* = 1). A wide range of AFO designs, construction and materials were used across included studies. AFO intervention(s) across studies included customised designs (*n* = 5 studies), prefabricated designs (*n* = 2 studies) or a comparison of both customised and prefabricated designs (*n* = 4 studies). The remaining four studies did not provide details regarding the fabrication of their AFO interventions. Detailed descriptions of the AFOs and testing protocols used are presented in Supporting Information S2. Seven studies reported providing participants with an AFO wear‐in period prior to testing, which ranged from 3 weeks to several years, four studies assessed the immediate effect of AFOs (i.e., no wear‐in period) and four studies did not report whether wear‐in time was provided. Five studies reported testing of comparator and intervention(s) conditions in a randomised order.

### 3.4 Quality assessment

**FIGURE 2:**
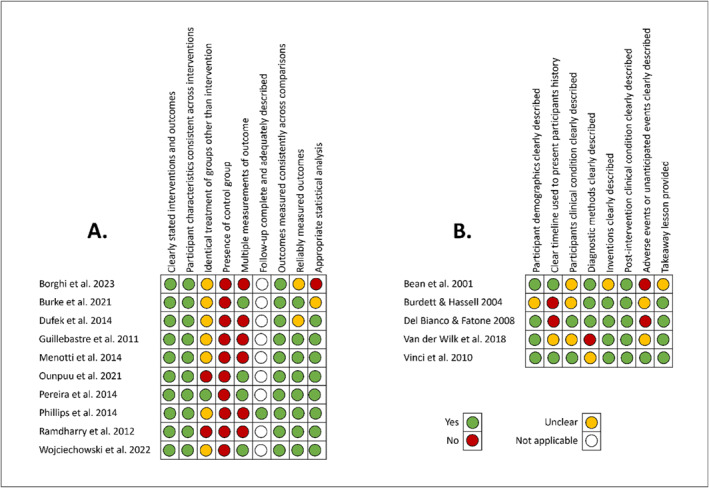
Quality assessment of included studies using JBI assessment tools. (A) Quasi‐experimental designs; (B) Case reports. JBI, Joanna Briggs Institute.

Results from the quality assessment are presented in Figure 2. Ten studies were assessed with the JBI checklist for quasi‐experimental studies (Figure 2A). All studies consistently provided clear descriptions of the interventions, participants' characteristics and outcome measures. However, deficiencies were noted in the lack of reporting on the consistency of treatments provided outside of the interventions and the lack of repeated assessments of the outcome measures. In examining the methodological quality of the five case reports (Figure 2B), it was noted that most of them provided a clear description of participant demographics, and clear descriptions of the intervention(s) and impact of the AFO(s) on post‐intervention outcomes. However, only two studies clearly described the case study's CMT characteristics, and only one study described adverse or unanticipated events as a result of the intervention(s).

### 3.5 Impact of AFOs on gait kinematics

Thirteen studies reported on the impact of AFOs on gait kinematics, including temporospatial parameters of gait (gait speed, stride length, step length, cadence, time in double and single limb support) and angular measurements associated with ankle, knee, hip and pelvis motion in sagittal and frontal planes. Statistical differences between comparator and AFO interventions were reported by eight studies with variable results (Table 2, Supporting Information S3).

**TABLE 2:** The impact of AFOs on gait kinematics (*n* = 13 studies).

Study	Interventions and comparators	Impact of AFOs on gait kinematics compared to comparator conditions
Borghi et al. 2023 [36]	**Comparator**: Barefoot	Both AFOs resulted in increased stride, speed, maximum ankle dorsiflexion during stance, and walking velocity as measured by a 10‐m walk test. Both AFOs resulted in decreased maximum ankle plantarflexion during swing, maximum knee flexion during swing, maximum hip flexion during swing, hip ROM during terminal swing, minimum knee flexion during single support and pelvis rotation. *Statistical differences between comparator and AFO interventions were not reported*.
	**Intervention 1**: Customised silicone AFO	
	**Intervention 2**: Customised botter AFO	
Burdett & Hassell 2004 [37]	**Comparator**: Shoes only	All AFOs resulted in an increased foot angle at maximum swing, foot angle at foot strike and foot angle at toe‐off. All AFOs resulted in decreased gait speed, plantarflexion angle at maximum swing, hip flexion angle at maximum swing, knee flexion angle at maximum swing, plantarflexion angle at foot strike, plantarflexion angle at toe‐off, hip flexion angle at toe‐off and knee flexion angle at toe‐off. *Statistical differences between comparator and AFO interventions were not reported*.
	**Intervention 1**: Customised solid ankle AFO	
	**Intervention 2**: Customised posterior leaf spring AFO	
	**Intervention 3**: Prefabricated AFO	
Del Bianco & Fatone 2008 [39]	**Comparator**: Shoes only	Both AFOs resulted in increased walking speed, stride length, cadence, step length, ankle angle at mid swing and peak hip flexion in stance. Both AFOs resulted in decreased step width, ankle range of motion during gait cycle, peak dorsiflexion in stance, minimum knee angle in late stance and peak hip extension. Total support decreased with Intervention 1 and increased with Intervention 2. *Statistical differences between comparator and AFO interventions were not reported*.
	**Intervention 1**: Customised posterior leaf spring AFO	
	**Intervention 2**: Customised silicone AFO	
Dufek et al. 2014 [40]	**Comparator**: Shoes only	The AFO resulted in statistically significant increases in walking velocity for six participants, cadence for six participants, stride length for seven participants and step length for seven participants (all *P* < 0.05). The AFO resulted in a statistically significant reduction in double support time for five participants (*P* < 0.05).
	**Intervention 1**: Customised carbon‐fibre composite AFO	
Guillesbastre et al. 2011 [41]	**Comparator**: Shoes with/without usual brace	Both AFOs resulted in a statistically significant increase in step length (*P* < 0.05). No statistically significant differences were found between comparator and AFO interventions for gait velocity, step time or percentage of gait cycle from heel off‐on, although all improved with AFOs.
	**Intervention 1**: Plastic AFO	
	**Intervention 2**: Elastic AFO	
Menotti et al. 2014 [42]	**Comparator**: Shoes only	No statistically significant differences were found between comparator and AFO for walking speed, step length, or step frequency, at either slow, comfortable or fast walking speeds (all *P* > 0.05).
	**Intervention 1**: Anterior elastic AFO	
Ounpuu et al. 2021 [43]	**Comparator**: Barefoot	The AFO resulted in statistically significant increases in step length, stride length, walking velocity, ankle angle at initial contact, peak ankle plantarflexion during swing, peak ankle plantarflexion during mid‐third of swing and foot progression angle (all *P* ≤ 0.001). The AFO resulted in statistically significant decreases in cadence, ankle range of motion and hip flexion (all *P* ≤ 0.001). No statistically significant differences were found between comparator and AFO for cycle time, peak ankle dorsiflexion during stance and time to peak ankle dorsiflexion during stance (all *P* > 0.05).
	**Intervention 1**: AFO	
Pereira et al. 2014 [44]	**Comparator**: Shoes only	The AFO resulted in a statistically significant reduction in stepping speed (*P* = 0.022) and ankle amplitude (*P* = 0.013). No statistically significant differences were found between comparator and AFO for hip movement amplitude or knee movement amplitude (all *P* > 0.05).
	**Intervention 1**: AFO	
Phillips et al. 2012 [45]	**Comparator**: Barefoot or shoes only	The AFOs resulted in statistically significant increases in stride length (*P* = 0.01), velocity (*P* = 0.006) and swing velocity (*P* = 0.0032) and reductions in stance time (*P* = 0.02). No statistically significant differences were found between comparator and AFOs for stance time, swing time, stride length, stride time or cadence (all *P* > 0.05).
	**Intervention 1**: Customised silicone AFO	
	**Intervention 2**: Customised polypropylene AFO	
	**Intervention 3**: Prefabricated AFO	
Ramdharry et al., 2012 [46]	**Comparator**: Shoes only	Compared to the comparator, all AFOs resulted in a statistically significant increase in dorsiflexion angle at foot clearance (*P* < 0.005). Intervention 3 resulted in a statistically significant increase in double support time and Intervention 1 and 3 resulted in a statistically decrease in peak hip flexion angle compared to the comparator (all *P* < 0.005).
	**Intervention 1**: Prefabricated anterior elastic AFO	
	**Intervention 2**: Prefabricated push brace AFO	
	**Intervention 3**: Posterior leaf AFO	
Van der Wilk et al. 2018 [47]	**Comparator**: Participant's existing AFO	All AFO interventions increased ankle ROM during controlled plantarflexion and dorsiflexion. Intervention 1 reduced ankle ROM during powered plantarflexion and Interventions 2–4 increased ankle ROM during powered plantarflexion. *Statistical differences between comparator and AFO interventions were not reported*.
	**Intervention 1**: ADJUST AFO stiff	
	**Intervention 2**: ADJUST AFO stiff/flexible	
	**Intervention 3**: ADJUST AFO flexible	
	**Intervention 4**: ADJUST AFO flexible/stiff	
Vinci et al. 2010 [48]	**Comparator**: Shoes only	AFOs resulted in increased walking velocity and increased cadence. Changes in stance, step length and step width differed between right and left limbs. Intervention 2 reduced swing velocity and Intervention 1 increased swing velocity. *Statistical differences between comparator and AFO interventions were not reported*.
	**Intervention 1**: Customised silicone AFO	
	**Intervention 2**: Codivilla	
	**Intervention 3**: Soft footdrop insert	
Wojciechowski et al. 2022 [49]	**Comparator**: Shoes only	The AFOs resulted in a statistically significant increase in ankle dorsiflexion at initial contact and decrease in maximum ankle plantarflexion at push‐off (all *P* < 0.05). No statistically significant differences were found between comparator and AFOs for maximum ankle dorsiflexion during swing, normalised walking speed, normalised stride length, normalised cadence, maximum ankle dorsiflexion in stance, timing of maximum ankle dorsiflexion in stance, maximum ankle dorsiflexion in the last 1/3 of swing, foot progression angle at 25%, thigh foot angle at 25%, knee flexion at initial contact, maximum knee flexion in loading response, minimum knee flexion in stance, maximum knee flexion in swing, minimum hip flexion, maximum hip flexion in swing, hip flexion ROM in swing, maximum hip adduction in stance, maximum hip adduction in swing, hip rotation at 25%, maximum pelvic tilt, maximum pelvic obliquity in swing, pelvic obliquity ROM in swing and pelvic rotation at 25% (all *P* > 0.05)
	**Intervention 1**: Traditional AFO	
	**Intervention 2**: 3D printed replica AFO	
	**Intervention 3**: 3D printed redesigned AFO	

Abbreviations: AFO, ankle foot orthosis; ROM, range of motion.

Data were available to conduct meta‐analyses for pooled SMDs between AFO and control groups for eight kinematic outcome measures including four temporal and spatial parameters of gait (walking velocity, stride length, cadence and step length) and four sagittal plane joint motion parameters (maximum ankle dorsiflexion during stance, maximum ankle plantarflexion during swing, maximum knee flexion during swing and maximum hip flexion during swing).

**FIGURE 3:**
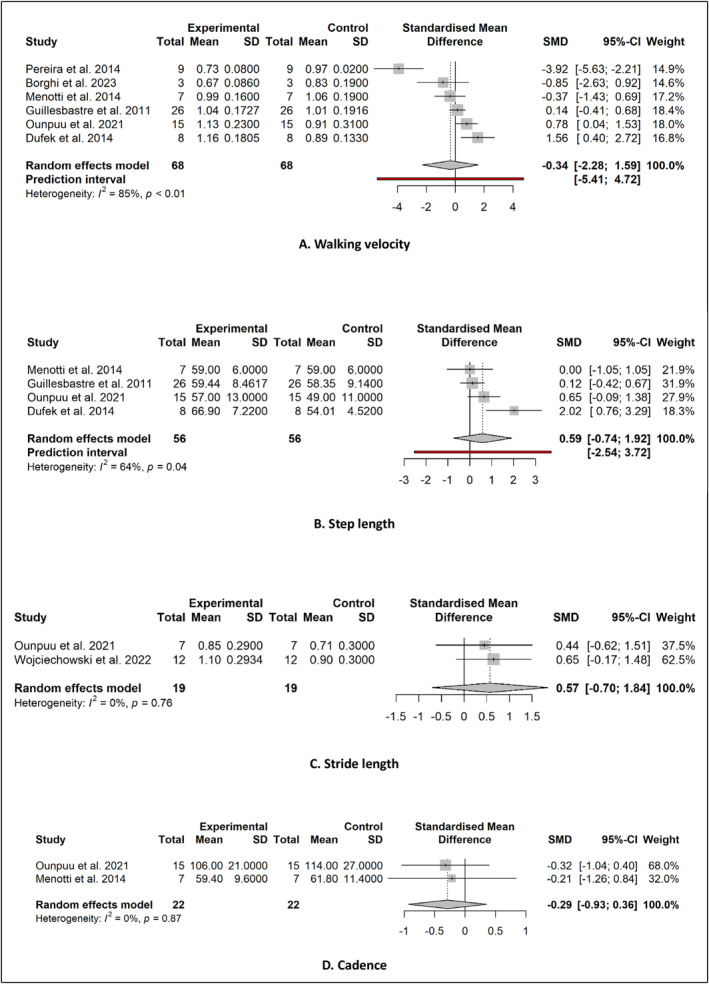
Pooled SMDs in temporospatial gait kinematics between AFOs and controls. (A) Walking velocity. (B) Step length. (C) Stride length. (D) Cadence. AFOs, Ankle‐foot orthoses; SMDs, standardised mean differences.

Forest plots of meta‐analyses for spatial and temporal gait kinematics are shown in Figure 3. Pooled data from six studies comparing AFO conditions to a control group showed a small and non‐significant effect of walking velocity in favour of the control groups (SMD −0.34; 95% CI −2.28–1.59, *P* = 0.667) (Figure 3A). Pooled data from two studies showed a non‐significant moderate effect for stride length (SMD 0.57, 95% CI −0.70–1.84, *P* = 0.110) (Figure 3B). Pooled data from two studies showed a small and non‐significant effect of cadence in favour of the control groups (SMD −0.29; 95% CI −0.93–0.36; *P* = 0.111) (Figure 3C). Pooled data from four studies showed a moderate non‐significant effect of step length in favour of the AFO conditions (SMD 0.59, 95% CI −0.74–1.92; *P* = 0.253) (Figure 3D).

**FIGURE 4:**
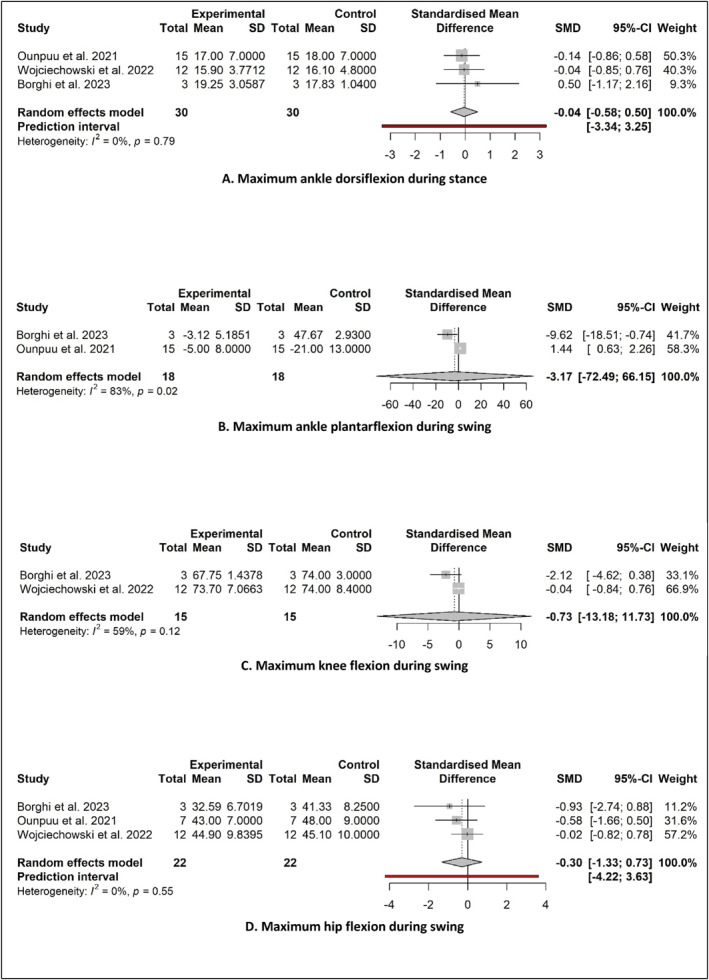
Pooled SMDs in ROM gait kinematics between control and AFOs. (A) Maximum ankle dorsiflexion during stance. (B) Maximum ankle plantarflexion during swing. (C) Maximum knee flexion during swing. (D) Maximum hip flexion during swing. AFOs, Ankle‐foot orthoses; ROM, range of motion; SMDs, standardised mean differences.

Figure 4 displays the forest plots for meta‐analyses of the four sagittal plane joint motion parameters. Pooled data from three studies comparing AFO conditions to a control group showed a small non‐significant effect of maximum ankle dorsiflexion during stance in favour of the control groups (SMD −0.04; 95% CI −0.58–0.51; *P* = 0.773) (Figure 4A). Pooled data from two studies showed a small non‐significant effect for maximum ankle plantarflexion during swing in favour of the AFO conditions (SMD −3.17; 95% CI −72.49–66.15; *P* = 0.665) (Figure 4B). Pooled data from two studies showed a moderate non‐significant effect for maximum knee flexion during swing in favour of the AFO conditions (SMD −0.73; 95% CI −13.18–11.73; *P* = 0. 594) (Figure 4C). Finally, pooled data, from three studies showed a small non‐significant effect for maximum hip flexion during swing in favour of the AFO conditions (SMD −0.30; 95% CI −1.33–0.73; *P* = 0.338) (Figure 4D).

### 3.6 Impact of AFOs on gait kinetics

Ten studies reported on the impact of AFOs on gait kinetics, including joint moments and power in the ankle, knee and hip, and ground reaction forces, oxygen consumption and energy cost. Statistical differences between comparator and AFO interventions were reported by four studies with variable results (Table 3, Supporting Information S4).

**TABLE 3:** The impact of AFOs on gait kinetics (*n* = 10 studies).

Study	Interventions and comparators	Impact of AFOs on gait kinetics compared to comparator conditions
Bean et al. 2001 [35]	**Comparator**: Non‐customised posterior leaf spring AFO	The AFO resulted in increased time to reach 60% peak oxygen consumption and time to reach 75% peak oxygen consumption. The AFO resulted in a decreased rate pressure product at 22.8 mL of O^2^/kg/min and rate pressure product at 17.5 mL of O^2^/kg/min. *Statistical differences between comparator and AFO interventions were not reported*.
	**Intervention 1**: Customised semi‐rigid AFO	
Borghi et al. 2023 [36]	**Comparator**: Barefoot	Both AFO conditions resulted in increased maximum pre‐swing ankle moments at push‐off, peak vertical GRF at push‐off and maximum hip power at push off. Intervention 1 resulted in reduced maximum ankle push‐off power and Intervention 2 increased maximum ankle push‐off power. *Statistical differences between comparator and AFO interventions were not reported*.
	**Intervention 1**: Customised silicone AFO	
	**Intervention 2**: Customised botter AFO	
Burdett & Hassell 2004 [37]	**Comparator**: Shoes only	Both AFO conditions resulted in lower torque generation for dorsiflexion and plantarflexion. Intervention 1 and Intervention 3 resulted in longer dorsiflexion torques after foot strike. During midstance and propulsion the comparator and Intervention 3 resulted in lower plantarflexion torque. *Statistical differences between comparator and AFO interventions were not reported*.
	**Intervention 1**: Customised solid ankle AFO	
	**Intervention 2**: Customised posterior leaf spring AFO	
	**Intervention 3:** Prefabricated AFO	
Del Bianco & Fatone 2008 [39]	**Comparator**: Shoes only	Both AFO conditions resulted in increased peak ankle plantarflexion moment in stance and first peak of vertical GRF. Both AFO conditions resulted in decreased moment corresponding to minimum knee angle in stance. Intervention 1 decreased peak ankle moment in loading response and Intervention 2 increased peak ankle moment in loading response and heel strike transient. *Statistical differences between comparator and AFO interventions were not reported*.
	**Intervention 1**: Customised posterior leaf spring AFO	
	**Intervention 2**: Customised silicone AFO	
Dufek et al., 2014 [40]	**Comparator**: Shoes only	The AFO resulted in greater ankle joint extensor moments, ankle joint power at the time of maximum support moments, and hip power absorption at the time of occurrence of the peak ankle joint moment. *Statistical differences between comparator and AFO interventions were not reported*.
	**Intervention 1**: Customised carbon‐fibre composite AFO	
Menotti et al. 2014 [42]	**Comparator**: Shoes only	The AFO resulted in a statistically significant increase in energy cost per unit of time and per unit of distance during fast‐paced walking (both *P* < 0.05). The AFO resulted in a statistically significant decrease in energy cost per unit of time and per unit of distance during comfortable‐paced walking (both *P* < 0.05). No statistically significant differences were found between comparator and AFO in energy cost per unit of time and per unit of distance during slow‐paced walking (both *P* > 0.05).
	**Intervention 1**: Anterior elastic AFO	
Ounpuu et al. 2021 [43]	**Comparator**: Barefoot	The AFO resulted in a significant increase in peak ankle plantar flexor moment (*P* = 0.001) and a significant decrease in peak ankle dorsiflexor moment during loading (*P* < 0.001). No statistically significant differences were found between comparator and AFO in peak ankle plantarflexor generation or peak hip power generation at toe off (both *P* > 0.05).
	**Intervention 1**: AFO	
Ramdharry et al. 2012 [46]	**Comparator**: Shoes only	Compared to the comparator, all AFOs resulted in a statistically significant increase in stiffness into dorsiflexion and stiffness into plantarflexion (*P* < 0.005). Intervention 3 resulted in a statistically significant reduction in peak ankle power generation during pre‐swing compared to the comparator (*P* < 0.05).
	**Intervention 1**: Prefabricated anterior elastic AFO	
	**Intervention 2**: Prefabricated push brace AFO	
	**Intervention 3**: Posterior leaf AFO	
Van der Wilk et al. 2018 [47]	**Comparator**: Participant's existing AFO	All AFO interventions resulted in a reduction in powered plantarflexion maximum power. Interventions 1 and 2 resulted in a reduction in powered plantarflexion maximum moment and Interventions 3 and 4 resulted in no change in powered plantarflexion maximum moment. *Statistical differences between comparator and AFO interventions were not reported*.
	**Intervention 1**: ADJUST AFO stiff	
	**Intervention 2**: ADJUST AFO stiff/flexible	
	**Intervention 3**: ADJUST AFO flexible	
	**Intervention 4**: ADJUST AFO flexible/stiff	
Wojciechowski et al. 2022 [49]	**Comparator**: Shoes only	Interventions 1 and 2 resulted in a significant reduction in maximum ankle dorsiflexor moment in loading response (*P* < 0.001) and a significant increase in maximum ankle plantarflexion moment (*P* < 0.05). No statistically significant differences were found between comparator and AFO conditions for maximum ankle power generation at midstance, maximum ankle power at push‐off, maximum knee flexor moment in single support, maximum extensor moment in stance, mean sagittal plane knee moment in stance or maximum hip abductor moment in terminal stance (*P* > 0.05).
	**Intervention 1**: Traditional AFO	
	**Intervention 2**: 3D printed replica AFO	
	**Intervention 3**: 3D printed redesigned AFO	

Abbreviations: 3D, three dimensional; AFO, ankle foot orthosis; GRF, ground reaction force.

**FIGURE 5:**
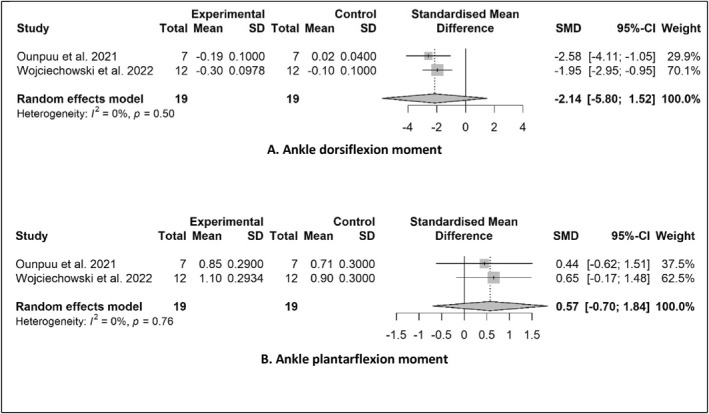
Pooled SMDs in gait kinetics between control and AFOs. (A) Ankle dorsiflexion moment. (B) Ankle plantarflexion moment. AFOs, Ankle‐foot orthoses; SMDs, standardised mean differences.

Data were available to conduct meta‐analyses for pooled SMDs between AFO and control groups for two kinetic outcome measures: maximum ankle dorsiflexion moment and maximum ankle plantarflexion moment (Figure 5). Pooled data from two studies comparing AFO conditions to a control group showed a non‐significant effect for ankle dorsiflexion moment (SMD −2.14; 95% CI −5.80–1.52; *P* = 0.085) (Figure 5A). Pooled data from the same two studies showed a non‐significant moderate effect for ankle plantarflexion moment (SMD 0.57; 95% CI −0.70–1.84; *P* = 0.110) (Figure 5B).

### 3.7 Impact of AFOs on postural stability/balance

Four studies reported on the impact of AFOs on postural stability/balance, including center of pressure velocity and displacement, and the time spent standing on various surfaces with eyes open and closed. Statistical differences between comparator and AFO conditions were reported by three studies (Table 4, Supporting Information S5). Due to the variability of data, meta‐analyses could not be conducted for these outcomes.

**TABLE 4:** The impact of AFOs on postural stability/balance (*n* = 4 studies).

Study	Interventions and comparators	Impact of AFOs on postural stability/balance compared to comparator conditions
Borghi et al. 2023 [36]	**Comparator**: Barefoot	Both AFO conditions resulted in reductions in mean CoP velocity and root mean square of CoP displacement. *Statistical differences between comparator and AFO interventions were not reported*.
	**Intervention 1**: Customised silicone AFO	
	**Intervention 2**: Customised botter AFO	
Burke et al. 2021 [38]	**Comparator**: Shoes only	No significant differences were found between comparator and AFO for time held with eyes open and eyes closed on firm surface and foam surface, and sway score with eyes open and eyes closed on firm surface and foam surface (all *P* > 0.05).
	**Intervention 1**: Dynamic carbon ground reaction AFO	
Guillesbastre et al. 2011 [41]	**Comparator**: Shoes with/without usual brace	Intervention 1 resulted in a statistically significant decrease in CoP trajectory area (*P* < 0.05). No significant differences were found between comparator and AFO conditions for CoP anterior posterior displacement amplitude, CoP medial lateral displacement amplitude, CoP anterior posterior displacement frequency or CoP medial lateral displacement frequency (all *P* > 0.05).
	**Intervention 1**: Plastic AFO	
	**Intervention 2**: Elastic AFO	
Pereira et al. 2014 [44]	**Comparator**: Shoes only	No significant differences were found between comparator and AFO for the Tinetti scale (an assessment of balance and gait) (*P* > 0.05).
	**Intervention 1**: AFO	

Abbreviations: AFO, ankle foot orthosis; CoP, center of pressure.

## 4 DISCUSSION

The results from this systematic review have shown that AFOs have a variable impact on several gait kinematics, kinetics and measures of postural stability and balance in people with CMT. Although pooled data from available studies demonstrated small‐to‐moderate effects of AFO use on a number of kinematic and kinetic parameters of gait, these effects were not statistically significant.

Although individually, many studies demonstrated statistically significant differences between AFO and non‐AFO testing conditions, when pooled, these differences were not evident. This may be explained by the variation in participant characteristics, CMT disease characteristics, AFO design and testing procedures. This also highlights the importance of device customisation as the impact that CMT has on foot and lower limb function is highly variable and standardised AFO designs may not be meeting the needs of every user [31]. The importance of AFO customisation has also been expressed by people with CMT, which can become challenging in the design of trials [32]. The recent advent of 3D‐printed AFOs increases the potential for customisation, and may better address needs relating to comfort and appearance for people with CMT [33]. Additionally, greater patient involvement in AFO design may also help to increase patient satisfaction.

CMT is a relatively rare condition, with an estimated overall prevalence of 40 cases per 100,000 [50], and as such, recruiting participants with CMT for research can be extremely challenging. This was reflected in the small sample sizes in the quasi‐experimental studies included in this review which ranged from 3 to 26. Sample size challenges are also the most cited reason for trial discontinuation and non‐publication of studies investigating rare conditions as they lack statistical power or fail to achieve the conventional levels of statistical significance [51]. We specifically chose to include case reports in the narrative summary of this review as they still contribute important insights that can continue to advance knowledge in this area [52, 53].

Although the within‐subject comparison designs used among the majority of included studies is an effective way of reducing sample size requirements by allowing the effects of both control and interventions to be tested on the same participants, carryover effects are challenging when testing the effects of more than one intervention. Most studies included in this review also involved none‐to‐minimal wear‐in time prior to testing and were therefore limited to testing the immediate effects of AFO use on gait. Further parallel‐group RCTs of the short‐ and long‐term impacts of AFO use in people with CMT are required. This is particularly important due to the reported physical and psychological discomfort associated with longer term AFO use among people with CMT [54], with one third reporting that their prescribed devices are uncomfortable or cause pain or skin irritation [12]. Dissatisfaction with device aesthetics, especially among females with CMT, also results in poor compliance with AFOs [55], which has been reported to range from 20% to 49% in people with CMT [15, 55]. Further research is required to assess both short‐ and long‐term effects of orthoses on not only gait but also on patient satisfaction and compliance.

This study has a number of strengths and limitations. Firstly, the review was conducted with a rigorous search strategy, and involvement of multiple authors at study screening, extraction and quality assessment stages. Few studies could be pooled for meta‐analysis due to the incomplete reporting of results, the wide variation in outcome measures used and the use of non‐parametric statistics, such as medians and ranges (rather than means and standard deviations). The included studies also had small sample sizes which are known to overestimate the true effect [33]. Despite adjusting for small sample size bias using the Hedges *g* correction and Knapp–Hartung adjustments to account for the few studies available for pooling, none of the meta‐analyses included more than 68 participants, so they may have been underpowered. The methodological quality of included studies also varied with an absence of true RCTs. Finally, there was notable heterogeneity in the AFO interventions used in the included studies, which differed in terms of material, manufacture, footplate length and mechanical characteristics. Although the aim of this review was not to explore whether the impact of AFOs on gait differed based on design features, these limitations highlight the need for further research involving adequate sample sizes to compare different AFO designs. This will allow optimal prescription algorithms to be established based on varying levels of gait impairment in people with CMT.

In conclusion, this systematic review and meta‐analysis has shown that AFOs do impact a number of gait and balance parameters. However, due to the small sample sizes and variability in participant characteristics, AFO designs and testing procedures adopted by the available studies, the effect of AFOs are not statistically significant when studies are pooled. This review has highlighted the importance of device customisation based on the individual needs of people with CMT and their degree of gait impairment.

## AUTHOR CONTRIBUTIONS

**Andrew Kim**: Data Curation; investigation; methodology; visualization; writing—original draft. **Mike Frecklington**: Conceptualization; data curation; methodology; supervision; writing—review & editing. **Adam Philps**: Conceptualization; writing—review & editing. **Sarah Stewart**: Conceptualization; data curation; formal analysis; methodology; supervision; visualization; writing—original draft; writing—review & editing.

## CONFLICT OF INTEREST STATEMENT

SS is on the Editorial Board for the Journal of Foot and Ankle Research. The other authors declare no competing interests.

## ETHICS STATEMENT

Ethical approval was not required for this analysis.

## Supporting information

Supporting Information S1

## Data Availability

The data that support the findings of this study are available in the supplementary material of this article.
