# Supplementary material for: The effect of ankle‐foot orthoses on gait characteristics in people with Charcot‐Marie‐Tooth disease: A systematic review and meta‐analysis
Source: J Foot Ankle Res. 2024 Sep 14;17(3):e70003. doi: 10.1002/jfa2.70003 (PMC11401480; doi:10.1002/jfa2.70003)
Supplement: Supplementary file 1 — Supporting Information S1 [file JFA2-17-e70003-s001.docx]

**SUPPORTING INFORMATION**

**Supporting Information 1.** Search terms used for electronic databases

| *Search terms used for* *CENTRAL (via Wiley)* | |
| --- | --- |
| #1 | MeSH descriptor: [Charcot‐Marie‐Tooth Disease] this term only |
| #2 | charcot:ti or charcot:ab |
| #3 | (hereditary near neuropathy):ti or (hereditary near neuropathy):ab |
| #4 | (hereditary near neuropathies):ti or (hereditary near neuropathies):ab |
| #5 | MeSH descriptor: [Hereditary Sensory and Motor Neuropathy] this term only |
| #6 | peroneal and atrophy |
| #7 | MeSH descriptor: [Muscular Atrophy, Adult Spinal] explode all trees |
| #8 | "spinal muscular atrophy" |
| #9 | distal and atrophy |
| #10 | #1 or #2 or #3 or #4 or #5 or #6 or #7 or #8 or #9 |
| #11 | MeSH descriptor: [Foot Orthoses] explode all trees |
| #12 | afo or brace* or bracing or orthotic* or orthoses OR orthosis OR splint* OR caliper OR leaf-spring |
| #13 | #11 or #12 |
| #14 | #10 and #13 |

| *Search terms used for* *MEDLINE and AMED (via Ovid)* | |
| --- | --- |
| #1 | Charcot-Marie-Tooth Disease/ |
| #2 | "Hereditary Sensory and Motor Neuropathy"/ |
| #3 | (charcot or (heredit$ adj5 neuropath$)).tw. |
| #4 | (peroneal and atroph$).tw. |
| #5 | muscular atrophy, spinal/ |
| #6 | (distal and atroph$).tw. |
| #7 | (inherit$ and neuropath$).tw. |
| #8 | or/1‐7 |
| #9 | exp "Orthotic Devices"/ |
| #10 | (afo or brace$ or bracing or orthotic$ or orthoses OR orthosis OR splint$ OR caliper OR leaf-spring).mp. |
| #11 | 9 or 10 |
| #12 | 8 and 11 |

| *Search terms used for* *CINAHL and SPORTDiscus (via EBSCO)* | |
| --- | --- |
| #1 | (MH "Charcot-Marie-Tooth Disease") |
| #2 | (MH "Neuropathies, Hereditary Motor and Sensory") |
| #3 | (charcot or (heredit* w/5 neuropath*)) |
| #4 | (peroneal and atrophy*) |
| #5 | (MM "Muscular Atrophy, Spinal") |
| #6 | (distal and atrophy*) |
| #7 | (inherit* and neuropath*) |
| #8 | or/1‐7 |
| #9 | (MH "Foot Orthoses+") |
| #10 | (afo or brace* or bracing or orthotic* or orthoses OR orthosis OR splint* OR caliper OR leaf-spring) |
| #11 | 9 or 10 |
| #12 | 8 and 11 |

| *Search terms used for* *Scopus* |
| --- |
| ("charcot marie tooth" OR "hereditary W/3 neuropath*" OR (peroneal AND atroph*) OR (distal AND atroph*) OR (inherit* AND neuropath* )) AND (orthotic OR orthosis OR orthoses OR brace OR bracing OR splint OR "leaf spring" OR caliper) |

| *Search terms used for* *PEDro* |
| --- |
| Charcot Marie Tooth |

| *Search terms used for* *Google Scholar* |
| --- |
| The first 100 records using the following search term: charcot marie tooth AND (afo OR orthoses OR orthosis OR orthotic OR brace OR bracing OR caliper OR leaf-spring) |

| **Supporting Information 2.** Intervention and protocol details | | | |
| --- | --- | --- | --- |
| **Study** | **Description of comparator and AFO conditions** | **AFO wear-in time and testing protocol used** | **Gait analysis protocol used** |
| Bean et al. 2001 | **Comparator**: Non-customised posterior leaf spring AFO (participants existing AFOs worn for 3 years)  **Intervention 1**: Customised semi-rigid AFO. Constructed from plastic. | One month wear-in time for Intervention 1 prior to testing. | Performed on treadmill at 3.0 miles/hour with grade increased by 1% every minute. |
| Borghi et al. 2023 | **Comparator**: Barefoot  **Intervention** **1**: Silicone AFO. Custom-molded with no joints and limited flexibility. Stiffness and angle of ankle at rest were adapted to each patient.  **Intervention** **2**: Botter AFO. Custom-molded with rear of base adapted to heel of shoe and forefoot pointing upwards. Posterior crossbow made of carbon and aramid fibres. Stiffness of forefoot and posterior crossbow and angle of ankle at rest were tailored to patients' weight and height. | Variable wear-in time prior to testing (multiple years) with at least one month wear-in time prior to testing with Intervention 2. | Performed on 10m Vicon System walkway (equipped with eight optoelectronic cameras, two force plates, and two video cameras) at self-selected walking speed. |
| Burdett & Hassell 2004 | **Comparator**: Shoes only. Participant's usual high-top athletics shoes.  **Intervention 1**: Customised solid ankle AFO. Constructed with 4.8mm co-polymer. Ankle set at neutral. Standard anterior trim lines and sulcus length foot-plate. Resistance to inversion/eversion. Worn in participants usual shoes with moderate rocker added for smoother toe-off.  **Intervention 2**: Customised posterior leaf spring AFO. Constructed from 4.8mm co-polymer with posterior reinforcement. Posterior trim lines. Ankle set at neutral and foot plate cut to sulcus length. Resisted plantarflexion with little resistance to dorsiflexion/inversion/eversion. Worn in participants usual shoes.  **Intervention 3:** Prefabricated AFO. Worn in participants usual shoes. Constructed with carbon and Kevlar. Consisting of foot plate, an anterior shell and lateral strut. | One month wear-in time prior to testing. | Performed using multiple instruments: a 10m walkway at self-selected walking speed; a treadmill set at a comfortable working speed of 0.54 m/sec and video recorded and analysed using Peak Performance Technologies 2D motion analysis system; an AMTI force plate to capture ankle torque and power. |
| Burke et al. 2021 | **Comparator**: Shoes only. Participant's own footwear.  **Intervention 1**: Dynamic carbon ground reaction AFO. Anterior tibial shell with short strut extending around instep to a footplate. Constructed with flexible carbon fibre material. Not custom fit for each participant. Worn with own shoes or shoes provided by orthotist. | Not reported. | NA |
| Del Bianco & Fatone 2008 | **Comparator**: Shoes only. Leather uppers and firm rubber soles.  **Intervention 1:** Customised posterior leaf spring AFO. Constructed from 3/16-inch co-polymer plastic. Proximal trim line positioned 25 mm distal to fibula head and medial and lateral trim lines 1.5cm anterior to medial malleolus. Sulcus-length foot plate. Worn in leather shoes.  **Intervention 2**: Customised silicone AFO. Constructed from silicone material (1/16 to 1/4 inch thickness). Wrapped around foot, ankle and lower leg with proximal trim line 28cm from floor and distal trim line proximal to metatarsal heads. Worn in leather shoes. | Nine-month wear-in time for Intervention 1 and 3 month wear-in time for Intervention 2. | Performed on a 10m walkway using the 8-camera digital real-time motion capture system embedded with six force-plates. |
| Dufek et al. 2014 | **Comparator**: Shoes only.  **Intervention 1**: Customised carbon-fibre composite AFO. Fabricated from multiple layers of bidirectional carbon, bidirectional carbon-Kevlar, and vacuum formed with epoxy matrix. Posterior spring brace wrapped around anterior leg with connecting strap. | At least ten-weeks wear-in time. | Performed on a 4.27m walkway at a self-selected comfortable walking speed, with dual fore platforms, and a 12 camera motion capture system. |
| Guillesbastre et al. 2011 | **Comparator**: Shoes with/without usual brace  **Intervention 1**: Plastic AFO. Worn with their own ordinary shoes.  **Intervention 2:** Elastic AFO. Worn with own ordinary shoes. | Testing order was randomised. | Performed on a 8.3m walkway (GAITRite) at a self-selected walking speed. |
| Menotti et al. 2014 | **Comparator:** Shoes only  **Intervention 1**: Anterior elastic AFO. Talolast. Constructed from an anterior polypropylene leaf fixed proximally with elastic-adjustable Velcro and distally underneath shoelaces. | Testing order was randomised. | Performed on an oval-shaped 23m walkway at three self-selected walking speeds (slow, comfortable, fast). |
| Ounpuu et al. 2021 | **Comparator**: Barefoot  **Intervention** **1:** AFO. Not standardised. Included solid AFO (n = 4 participants), posterior leaf spring AFO (n = 4 participants), floor reaction AFO (n = 3 participants), hinged AFO (n = 4). | No wear-in period. Immediate testing. | Performed using a 20m Vicon System walkway equipped with 12 cameras and three AMTI force platforms, at a self-selected walking speed. |
| Pereira et al. 2014 | **Comparator**: Shoes only. Participants usual shoes.  **Intervention** **1**: AFO. Design and standardisation not reported. | No wear-in period. Immediate testing. | Performed on a 3m walkway at a self-selected usual walking speed with three interconnected infrared cameras. |
| Phillips et al. 2012 | **Comparator**: Barefoot or shoes only (if participant could not walk barefoot).  **Intervention 1**: Silicone AFO. Custom-made from silicone to a cast of the participant's foot/ankle. Designed to restrict plantarflexion during swing.  **Intervention 2**: Polypropylene AFO. Custom-made from polypropylene to a cast of the participant's foot/ankle.  **Intervention 3**: Prefabricated AFO. Ligaflex™ stock fabric AFO with two elasticised straps that increase dorsiflexion. | Testing order randomised.  Three-week wear in time with one-week washout period in between. | Not reported. |
| Ramdharry et al., 2012 | **Comparator:** Shoes only. Participant's own flat lace-up sport or casual shoe.  **Intervention 1:** Prefabricated anterior elastic AFO. Cuff worn around ankle with detachable anterior elastic strap and plastic flange attached to laces of shoe.  **Intervention 2:** Prefabricated push brace AFO. Constructed from preformed foam covering medial, posterior and lateral ankle and extending under heel. Reinforced by three elastic straps.  **Intervention 3:** Posterior leaf AFO. Multifit Achilles drop foot orthosis. Variant of posterior leaf AFO made of plastic polymer with half foot plate, cut-out heel section, and adjustable back stem extending to calf. Allows ankle to move into dorsiflexion during stance. | Testing order randomised.  No wear-in period. Immediate testing. | Performed on a customised walkway containing 2 embedded force plates. |
| Van der Wilk et al. 2018 | **Comparator:** Solid polypropylene dorsal AFOs with full-length foot plate. Participant's own existing AFOs worn for 22 years.  **Intervention 1:** ADJUST AFO stiff. Consists of two leaf-spring hinges independently controlling plantarflexion and dorsiflexion stiffness. Stiff medial and lateral spring.  **Intervention 2:** ADJUST AFO stiff/flexible. Same as above but stiff medial and flexible lateral spring.  **Intervention 3**: ADJUST AFO flexible. Same as above but flexible medial and lateral springs.  **Intervention 4**: ADJUST AFO flexible/stiff. Same as above but flexible medial and stiff lateral spring. | No wear-in time. Immediate testing. | Performed on a treadmill at a comfortable walking speed of 0.8m/sec using a infrared motion-capturing system with 10 infrared cameras. The treadmill was embedded with two force plates. |
| Vinci et al. 2010 | **Comparator**: Shoes only. With some plantar rearfoot and lateral wedges.  **Intervention 1**: Customised silicone AFO. Soft ankle with a reinforced front.  **Intervention 2**: Codivilla AFO. Constructed from two 15 x 1.2mm chromasteel springs fixed to a custom-made ankle boot by a proper square and a throttle bearing padded with soft material.  **Intervention 3**: Soft footdrop insert. Constructed from 10.5 x 30 x 0.5 cm sheet of reticulated polyoelphinic foam attached to mid-calf booths with a resistant raised-tip and plastic sole. | Three-year wear in time prior to testing. | Performed on a 10m walkway with two force platforms and eight infrared cameras. |
| Wojciechowski et al. 2022 | **Comparator:** Shoes only. All Dunlop Volley.  **Intervention 1:** Traditional AFO. Prescribed based on clinical indication and customised in propylene based on plaster casting methods. Included bilateral leaf spring (n = 4), bilateral hinged (n = 6), bilateral solid with supramalleolar insert (n = 1), right side hinged and left side solid with supramalleolar insert (n = 1).  **Intervention 2:** 3D printed replica AFO. Same design as traditional AFO but computer aided designs manufactured using material extrusion in Nylon 12. A 100% infill, 1.178mm layer thickness, and SR-110 dissolvable support material used.  **Intervention 3:** 3D printed redesigned AFO. Manufactured as Intervention 2, but personalised to each participant through design features aimed to reduce mass including posterior aperture, graduations in thickness, clasp attachments for straps and removable hinges. | Comparator tested first then testing order of interventions randomised. | Performed on an 8m Vicon system walkway imbedded with three AMTI force plates and with eight cameras. |

| ***Supporting Information 3.*** *Impact of AFOs on gait kinematics* | |
| --- | --- |
| **Outcome** | **Differences between control and intervention groups** |
| **Borghi et al. 2023** | |
| Stride (% of gait cycle) | Control (Barefoot): mean 60.67 (SD 6.43)  Intervention 1 (Silicone AFO): mean 77.00 (SD 1.41)  Intervention 2 (Botter AFO): mean 74.33 (SD 5.03)  Statistical differences between groups not reported |
| Speed (% of gait cycle) | Control (Barefoot): mean 50.0 (SD 13.53)  Intervention 1 (Silicone AFO): mean 60.00 (SD 9.90)  Intervention 2 (Botter AFO): mean 62.00 (SD 11.00)  Statistical differences between groups not reported |
| Maximum ankle dorsiflexion during stance (˚) | Control (Barefoot): mean 17.83 (SD 1.04)  Intervention 1 (Silicone AFO): mean 18.50 (SD 1.41)  Intervention 2 (Botter AFO): mean 20.00 (SD 4.44)  Statistical differences between groups not reported |
| Maximum ankle plantarflexion during swing (˚) | Control (Barefoot): mean 47.67 (SD 2.93)  Intervention 1 (Silicone AFO): mean -7.25 (SD 1.77)  Intervention 2 (Botter AFO): mean 1.00 (SD 3.61)  Statistical differences between groups not reported |
| Maximum knee flexion during swing (˚) | Control (Barefoot): mean 74.00 (SD 3.00)  Intervention 1 (Silicone AFO): mean 68.00 (SD 1.41)  Intervention 2 (Botter AFO): mean 67.50 (SD 1.73)  Statistical differences between groups not reported |
| Maximum hip flexion during swing (˚) | Control (Barefoot): mean 41.33 (SD 8.25)  Intervention 1 (Silicone AFO): mean 35.50 (SD 9.19)  Intervention 2 (Botter AFO): mean 29.67 (SD 1.53)  Statistical differences between groups not reported |
| Hip ROM during terminal swing (˚) | Control (Barefoot): mean 15.50 (SD 3.04)  Intervention 1 (Silicone AFO): mean 9.00 (SD 6.36)  Intervention 2 (Botter AFO): mean 8.17 (SD 4.25)  Statistical differences between groups not reported |
| Minimum knee flexion during single support (˚) | Control (Barefoot): mean 3.83 (SD 7.77)  Intervention 1 (Silicone AFO): mean -5.75 (SD 4.60)  Intervention 2 (Botter AFO): mean -6.00 (SD 5.41)  Statistical differences between groups not reported |
| Pelvis rotation (˚) | Control (Barefoot): mean 33.83 (SD 12.09)  Intervention 1 (Silicone AFO): mean 25.25 (SD 17.32)  Intervention 2 (Botter AFO): mean 19.17 (SD 14.15)  Statistical differences between groups not reported |
| 2-minute walk test (m) | Control (Barefoot): mean 145.0 (SD 21.2)  Intervention 1 (Silicone AFO): mean 113.5 (SD 58.7)  Intervention 2 (Botter AFO): mean 163.3 (SD 50.3)  Statistical differences between groups not reported |
| 10-meter walk test (sec) | Control (Barefoot): mean 8.3 (SD 1.9)  Intervention 1 (Silicone AFO): mean 6.9 (SD 1.3)  Intervention 2 (Botter AFO): mean 6.6 (SD 0.6)  Statistical differences between groups not reported |
| **Burdett & Hassell 2004** | |
| Gait speed (m/sec) | Control (Shoes only): 1.09 Intervention 1 (Solid ankle): 1.04 Intervention 2 (Posterior leaf spring AFO): 1.01 Intervention 3 (Prefabricated AFO): 1.01  Statistical differences between groups not applicable |
| Plantarflexion angle at maximum swing (˚) | Control (Shoes only): 23 Intervention 1 (Solid ankle AFO): 1.5 Intervention 2 (Posterior leaf spring AFO): 0.5 Intervention 3 (Prefabricated AFO): 16  Statistical differences between groups not applicable |
| Foot angle at maximum swing (relative to the horizontal, toe higher than heel is a positive angle) (˚) | Control (Shoes only): -70 Intervention 1 (Solid ankle AFO): -48.5 Intervention 2 (Posterior leaf spring AFO): -48 Intervention 3 (Prefabricated AFO): -64.5  Statistical differences between groups not applicable |
| Hip flexion angle at maximum swing (angle of thigh relative to vertical) (˚) | Control (Shoes only): 27 Intervention 1 (Solid ankle AFO): 25 Intervention 2 (Posterior leaf spring AFO): 25.5 Intervention 3 (Prefabricated AFO): 27  Statistical differences between groups not applicable |
| Knee flexion angle at maximum swing (˚) | Control (Shoes only): 66.5 Intervention 1 (Solid ankle AFO): 59.5 Intervention 2 (Posterior leaf spring AFO): 64 Intervention 3 (Prefabricated AFO): 65  Statistical differences between groups not applicable |
| Plantarflexion angle at foot strike (˚) | Control (Shoes only): 3 Intervention 1 (Solid ankle AFO): -7.5  Intervention 2 (Posterior leaf spring AFO): -2.5 Intervention 3 (Prefabricated AFO): 3  Statistical differences between groups not applicable |
| Foot angle at foot strike (relative to the horizontal, toe higher than heel is a positive angle) (˚) | Control (Shoes only): 2 Intervention 1 (Solid ankle AFO): 9 Intervention 2 (Posterior leaf spring AFO): 8 Intervention 3 (Prefabricated AFO): 3  Statistical differences between groups not applicable |
| Plantarflexion angle at toe-off (˚) | Control (Shoes only): 20 Intervention 1 (Solid ankle AFO): -1 Intervention 2 (Posterior leaf spring AFO): -3 Intervention 3 (Prefabricated AFO): 12.5  Statistical differences between groups not applicable |
| Foot angle at toe-off (relative to the horizontal, toe higher than heel is a positive angle) (˚) | Control (Shoes only): -69 Intervention 1 (Solid ankle AFO): -45.5 Intervention 2 (Posterior leaf spring AFO): -46 Intervention 3 (Prefabricated AFO): -60.5  Statistical differences between groups not applicable |
| Hip flexion angle at toe-off (angle of thigh relative to vertical) (˚) | Control (Shoes only): 64.5 Intervention 1 (Solid ankle AFO): 52 Intervention 2 (Posterior leaf spring AFO): 56 Intervention 3 (Prefabricated AFO): 57.5  Statistical differences between groups not applicable |
| Knee flexion angle at toe-off (˚) | Control (Shoes only): 16 Intervention 1 (Solid ankle AFO): 6 Intervention 2 (Posterior leaf spring AFO): 9 Intervention 3 (Prefabricated AFO): 10 |
| **Del Bianco & Fatone 2008** | |
| Speed (m/s) | Control (Shoes only): 0.78  Intervention 1 (Posterior leaf spring AFO): 1.09  Intervention 2 (Silicone AFO): 0.93  Statistical differences between groups not applicable |
| Stride length (cm) | Control (Shoes only): 102.6  Intervention 1 (Posterior leaf spring AFO): 113.6  Intervention 2 (Silicone AFO): 112.8  Statistical differences between groups not applicable |
| Cadence (steps/min) | Control (Shoes only): 91  Intervention 1 (Posterior leaf spring AFO): 97.9  Intervention 2 (Silicone AFO): mean 99  Statistical differences between groups not applicable |
| Step width (cm) | Control (Shoes only): 22.1  Intervention 1 (Posterior leaf spring AFO): 19.4  Intervention 2 (Silicone AFO): 21.1  Statistical differences between groups not applicable |
| Step length (cm) | Control (Shoes only): 51.1  Intervention 1 (Posterior leaf spring AFO): 66.85  Intervention 2 (Silicone AFO): 56.2  Statistical differences between groups not applicable |
| Total support time (% gait cycle) | Control (Shoes only): 67.15  Intervention 1 (Posterior leaf spring AFO): 65.8  Intervention 2 (Silicone AFO): 67.95  Statistical differences between groups not applicable |
| Ankle range of motion during gait cycle (˚) | Control (Shoes only): 30.4  Intervention 1 (Posterior leaf spring AFO): mean 18.35  Intervention 2 (Silicone AFO): 24.8  Statistical differences between groups not applicable |
| Peak dorsiflexion in stance (˚) | Control (Shoes only): 20.85  Intervention 1 (Posterior leaf spring AFO): 18.05  Intervention 2 (Silicone AFO): 18.5  Statistical differences between groups not applicable |
| Ankle angle at mid swing (˚) (dorsiflexion +/plantarflexion -) | Control (Shoes only): -7.45  Intervention 1 (Posterior leaf spring AFO): mean 5.35  Intervention 2 (Silicone AFO): -1.55  Statistical differences between groups not applicable |
| Minimum knee angle in late stance (˚) (flexion +/extension -) | Control (Shoes only): 0.5  Intervention 1 (Posterior leaf spring AFO): mean -6.55  Intervention 2 (Silicone AFO): -6.85  Statistical differences between groups not applicable |
| Peak hip flexion in stance (˚) | Control (Shoes only): 26.2  Intervention 1 (Posterior leaf spring AFO): 31.4  Intervention 2 (Silicone AFO): 29.25  Statistical differences between groups not applicable |
| Peak hip extension (˚) | Control (Shoes only): -10.9  Intervention 1 (Posterior leaf spring AFO): -13.4  Intervention 2 (Silicone AFO): -12.55  Statistical differences between groups not applicable |
| **Dufek et al. 2014** | |
| Walking velocity (cm/s) | Control (Shoes only): mean 89.44 (SD 13.30) Intervention 1 (Carbon-fibre AFO): mean 115.58 (SD 18.05)  Six participants had significantly faster velocity with AFO (P<0.05) |
| Cadence (hz) | Control (Shoes only): mean 0.92 (SD 0.15) Intervention 1 (Carbon-fibre AFO): mean 1.19 (SD 0.21)  Six participants had significantly higher cadence with AFO (P<0.05) |
| Left stride length (cm) | Control (Shoes only): mean 108.64 (SD 8.09)  Intervention 1 (Carbon-fibre AFO): mean 131.89 (SD 13.85)  Seven participants had significantly greater stride lengths with AFO (P<0.05) |
| Right stride length (cm) | Control (Shoes only): mean 108.46 (SD 8.20) Intervention 1 (Carbon-fibre AFO): mean 131.99 (SD 14.26)  Seven participants had significantly greater stride length with AFO (P<0.05) |
| Left step length (cm) | Control (Shoes only): mean 54.21 (SD 5.62) Intervention 1 (Carbon-fibre AFO): mean 65.86 (SD 7.72)  Seven participants had significantly greater step lengths with AFO (P<0.05) |
| Right step length (cm) | Control (Shoes only): mean 543.81 (SD 3.42) Intervention 1 (Carbon-fibre AFO):mean 67.91 (SD 6.72)  Seven participants had significantly greater step length with AFO (P<0.05) |
| Double support time (% gait cycle) | Control (Shoes only): Not reported Intervention 1 (Carbon-fibre AFO): Not reported  Five participants had significantly reduced double support time with AFO (P<0.05) |
| **Guillesbastre et al. 2011** | |
| Gait velocity (cm/s) | Control (Shoes +/- usual brace): mean 101.45 (SD 19.16)  Intervention 1 (Plastic AFO): mean 103.19 (SD 17.63)  Intervention 2 (Elastic AFO): mean 105.15 (SD 17.20)  No significant differences between groups |
| Step length (cm) | Control (Shoes +/- usual brace): mean 58.35 (SD 9.14)  Intervention 1 (Plastic AFO): mean 59.54 (SD 8.49)  Intervention 2 (Elastic AFO): mean 59.33 (SD 8.60)  Significantly lower in own shoes vs plastic AFO and lower in own shoes vs elastic AFO (P<0.05) |
| Step time (s) | Control (Shoes +/- usual brace): mean 0.58 (SD 0.05)  Intervention 1 (Plastic AFO): mean 0.58 (SD 0.04)  Intervention 2 (Elastic AFO): mean 0.57 (SD 0.04)  No significant differences between groups |
| Coefficients of variation step length | Control (Shoes +/- usual brace): mean 4.33 (SD 2.28)  Intervention 1 (Plastic AFO): mean 3.93 (SD 1.93)  Intervention 2 (Elastic AFO): mean 3.69 (SD 1.82)  No significant differences between groups |
| Coefficients of variation step time | Control (Shoes +/- usual brace): mean 3.72 (SD 1.64)  Intervention 1 (Plastic AFO): mean 3.53 (SD 1.83)  Intervention 2 (Elastic AFO): mean 3.52 (SD 1.25)  No significant differences between groups |
| Heel off-on (% gait cycle) | Control (Shoes +/- usual brace): mean 5.60 (SD 3.12)  Intervention 1 (Plastic AFO): mean 4.96 (SD 3.21)  Intervention 2 (Elastic AFO): mean 5.49 (SD 3.69)  No significant differences between groups |
| **Menotti et al. 2014** | |
| Walking speed at slow pace (m/s) | Control (Shoes only): mean 0.82 (SD 0.12)  Intervention 1 (Anterior elastic AFO): mean 0.78 (SD 0.12)  No significant differences between groups |
| Walking speed at comfortable pace (m/s) | Control (Shoes only): mean 1.03 (SD 0.19)  Intervention 1 (Anterior elastic AFO): mean 0.99 (SD 0.16)  No significant differences between groups |
| Walking speed at fast pace (m/s) | Control (Shoes only): mean 1.30 (SD 0.18)  Intervention 1 (Anterior elastic AFO): mean 1.32 (SD 0.16)  No significant differences between groups |
| Step length at slow pace (m) | Control (Shoes only): mean 0.53 (SD 0.05)  Intervention 1 (Anterior elastic AFO): mean 0.52 (SD 0.04)  No significant differences between groups |
| Step length at comfortable pace (m) | Control (Shoes only): mean 0.59 (SD 0.06)  Intervention 1 (Anterior elastic AFO): mean 0.59 (SD0.06)  No significant differences between groups |
| Step length at fast pace (m) | Control (Shoes only): mean 0.66 (SD 0.06)  Intervention 1 (Anterior elastic AFO): mean 0.68 (SD 0.06)  No significant differences between groups |
| Step frequency at slow pace (steps/s) | Control (Shoes only): mean 92.09 (SD 7.12)  Intervention 1 (Anterior elastic AFO): mean 91.69 (SD 11.17)  No significant differences between groups |
| Step frequency at comfortable pace (steps/s) | Control (Shoes only): mean 104.19 (SD 11.60)  Intervention 1 (Anterior elastic AFO): mean 109.75 (SD 12.78)  No significant differences between groups |
| Step frequency at fast pace (steps/s) | Control (Shoes only): mean 117.36 (SD 11.68)  Intervention 1 (Anterior elastic AFO): mean 116.81 (SD 24.08)  No significant differences between groups |
| **Ounpuu et al. 2021** | |
| Step length (m) | Control (Barefoot): mean 0.49 (SD 0.11)  Intervention 1 (AFO): mean 0.57 (SD 0.13)  Significant increase in step length with AFO (P=0.001) |
| Stride length (m) | Control (Barefoot): mean 0.96 (SD 0.22)  Intervention 1 (AFO): mean 1.13 (SD 0.23)  Significant increase in stride length with AFO (P=0.001) |
| Cycle time (s) | Control (Barefoot): mean 1.15 (SD 0.48)  Intervention 1 (AFO): mean 1.20 (SD 0.42)  No significant difference between groups |
| Cadence (steps/min) | Control (Barefoot): mean 114 (SD 27)  Intervention 1 (AFO): mean 106 (SD 21)  Significant decrease in cadence with AFO (P=0.001) |
| Walking velocity (m/sec) | Control (Barefoot): mean 0.91 (SD 0.31)  Intervention 1 (AFO): mean 1.13 (SD 0.23)  Significant increase in walking velocity with AFO (P=0.001) |
| Ankle angle at initial contact (˚) | Control (Barefoot): mean -10 (SD 7)  Intervention 1 (AFO): mean 1 (SD 6)  Significant increase in ankle angle with AFO (P=0.0001) |
| Peak ankle dorsiflexion during stance (˚) | Control (Barefoot): mean 18 (SD 7)  Intervention 1 (AFO): mean 17 (SD 7)  No significant difference between groups |
| Time to peak ankle dorsiflexion during stance (% gait cycle) | Control (Barefoot): mean 47 (SD 14)  Intervention 1 (AFO): mean 51 (SD 9)  No significant difference between groups |
| Peak ankle plantarflexion during swing (˚) | Control (Barefoot): mean -21 (SD 13)  Intervention 1 (AFO): mean -5 (SD 8)  Significant increase in peak ankle plantarflexion with (P=0.0001) |
| Peak ankle plantarflexion during mid 1/3 of swing (˚) | Control (Barefoot): mean -11 (SD 10)  Intervention 1 (AFO): mean 0 (SD 5)  Significant increase in peak ankle plantarflexion with AFO (P=0.0001) |
| Ankle range of motion (˚) | Control (Barefoot): mean 39 (SD 13)  Intervention 1 (AFO): mean 27 (SD 9)  Significant decrease in ankle ROM with AFO (P<0.0001) |
| Foot progression angle (˚) | Control (Barefoot): mean -13 (SD 15)  Intervention 1 (AFO): mean -4 (SD 16)  Significant increase in foot progression angle with AFO (P=0.001) |
| Peak hip flexion during swing (˚) | Control (Barefoot): mean 48 (SD 9)  Intervention 1 (AFO): mean 43 (SD 7)  Significant decrease in hip flexion with AFO (P=0.001) |
| **Pereira et al. 2014** | |
| Stepping speed (m/s) | Control (Shoes only): mean 0.97 (SD 0.02)  Intervention 1 (AFO): mean 0.73 (SD 0.08)  Significantly reduced stepping speed with AFO (P=0.022) |
| Hip movement amplitude (˚) | Control (Shoes only): mean 28 (SD 12)  Intervention 1 (AFO) : mean 26 (SD 1.4)  No significant difference between groups |
| Knee movement amplitude (˚) | Control (Shoes only): mean 36 (SD 0.5)  Intervention 1 (AFO): mean 35 (SD 1.6)  No significant difference between groups |
| Ankle movement amplitude (˚) | Control (Shoes only): mean 41 (SD 3.4)  Intervention 1 (AFO): mean 19 (SD 0.6)  Significant reduction in ankle amplitude with AFO (P=0.013) |
| **Phillips et al. 2012** | |
| Stance time (ms) | Control (Barefoot or shoes only): median 923 (IQR 848–1019)  Intervention 1 (Silicone AFO): median 827 (IQR 743–912)  Intervention 2 (Polypropylene AFO): median 795 (IQR 762–866)  No significant differences between groups |
| Stance time (% stride) | Control (Barefoot or shoes only): median 67.2 (IQR 64.5–68.4)  Intervention 1 (Silicone): median 65.2 (IQR 62.7–67.3)  Intervention 2 (Polypropylene AFO): median 65.2 (IQR 62.0–67.1)  AFOs were significantly different from control (P=0.02) |
| Swing time (ms) | Control (Barefoot or shoes only): median 442 (IQR 396–522)  Intervention 1 (Silicone AFO): median 431 (IQR 390–469)  Intervention 2 (Polypropylene AFO): median 436 (IQR 426–471)  No significant differences between groups. |
| Swing time (% stride) | Control (Barefoot or shoes only): median 32.9 (IQR 31.6–35.5)  Intervention 1 (Silicone AFO): median 34.9 (IQR 32.7–37.3)  Intervention 2 (Polypropylene AFO): median 34.8 (IQR 32.9–38.0)  No significant differences between groups |
| Stride time (ms) | Control (Barefoot or shoes only): median 1372 (IQR 1250-1546)  Intervention 1 (Silicone AFO): median 1248 (IQR 1155-1336)  Intervention 2 (Polypropylene AFO): median 1252 (IQR 1160-1294)  No significant differences between groups |
| Cadence (step min-1) | Control (Barefoot or shoes only): median 87.9 (IQR 77.8-96.2)  Intervention 1 (Silicone AFO): median 96.4 (IQR 90.1-104.5)  Intervention 2 (Polypropylene AFO): median 95.9 (IQR 93.0-103.4)  No significant differences between groups |
| Stride length (mm) | Control (Barefoot or shoes only): median 988 (IQR 885–1199  Intervention 1 (Silicone AFO): median 1065 (IQR 954–1455)  Intervention 2 (Polypropylene AFO): median 1150 (IQR 954–1455)  AFOs were significantly different from control (P=0.01) |
| Velocity (ms-1) | Control (Barefoot or shoes only): median 0.79 (IQR 0.56–0.84)  Intervention 1 (Silicone AFO): median 0.88 (IQR 0.71–1.12)  Intervention 2 (Polypropylene AFO): median 0.96 (IQR 0.75–1.18)  AFOs were significantly different from control (P=0.006) |
| Swing velocity (ms-1) | Control (Barefoot or shoes only): median 1.96 (IQR 1.48–2.09)  Intervention 1 (Silicone AFO): median 2.12 (IQR 1.87–2.55)  Intervention 2 (Polypropylene AFO): median 2.32 (IQR 1.84–2.61)  AFOs were significantly different from control (P=0.032) |
| **Ramdharry et al., 2012** | |
| Double support (% gait cycle) | Comparator (Shoes only): mean 15.32 (SE 3.01)  Intervention 1 (Anterior elastic AFO): mean 15.68 (SE 0.46)  Intervention 2 (Push brace AFO): mean 16.05 (SE 0.45)  Intervention 3 (Posterior leaf AFO): mean 16.25 (SE 0.42)  Intervention 3 was significantly greater than shoes only (P<0.05) |
| Dorsiflexion angle at foot clearance(˚) | Comparator (Shoes only): mean -3.86 (SE 62.04)  Intervention 1 (Anterior elastic AFO): mean 1.23 (SE 0.56)  Intervention 2 (Push brace AFO): mean 0.46 (SE 1.26)  Intervention 3 (Posterior leaf AFO): mean 1.69 (SE 1.35)  All orthoses were significantly greater than shoes only (P<0.05) |
| Peak hip flexion angle (˚) | Comparator (Shoes only): mean 36.93 (SE 2.09)  Intervention 1 (Anterior elastic AFO): mean 34.89 (SE 1.58)  Intervention 2 (Push brace AFO): mean 33.36 (SE 2.04)  Intervention 3 (Posterior leaf AFO): mean 35.51 (SE 1.94)  Intervention 1 and 3 were significantly lower than shoes only (P<0.05) |
| **Van der Wilk et al. 2018** | |
| Ankle ROM during controlled plantarflexion (initial contact until maximum plantarflexion) (˚) | Patients own AFO: 1  Intervention 1 (ADJUST AFO stiff): 6 Intervention 2 (ADJUST AFO stiff/flexible): 7  Intervention 3 (ADJUST AFO flexible): 8  Intervention 3 (ADJUST AFO flexible): 10  Statistical differences between groups not reported |
| Ankle ROM during controlled dorsiflexion (maximum plantarflexion until maximum dorsiflexion) (˚) | Patients own AFO: 21  Intervention 1 (ADJUST AFO stiff): 28  Intervention 2 (ADJUST AFO stiff/flexible): 58  Intervention 3 (ADJUST AFO flexible): 31  Intervention 4 ADUGST AFO flexible/stiff: 33  Statistical differences between groups not reported |
| Ankle ROM during powered plantarflexion (maximum dorsiflexion until toe off) (˚) | Patients own AFO: 19  Intervention 1 (ADJUST AFO stiff): 17  Intervention 2 (ADJUST AFO stiff/flexible): 50  Intervention 3 (ADJUST AFO flexible): 23  Intervention 4 (ADJUST AFO flexible): 22  Statistical differences between groups not reported |
| **Vinci et al. 2010** | |
| Walking velocity (m/s) | Control (Shoes only): 0.64 Intervention 1 (Silicone AFO): Not reported  Intervention 2 (Codivilla): 0.69  Intervention 3 (Soft foot insert): 0.8  Statistical differences between groups not applicable. |
| Right Swing velocity (m/s) | Control (Shoes only): 1.92 Intervention 1 (Silicone AFO): Not reported  Intervention 2 (Codivilla): 1.85  Intervention 3 (Soft foot insert): 2.15  Statistical differences between groups not applicable |
| Left Swing velocity (m/s) | Control (Shoes only): 1.54 Intervention 1 (Silicone AFO): Not reported  Intervention 2 (Codivilla): 1.69  Intervention 3 (Soft foot insert): 2.05  Statistical differences between groups not applicable |
| Cadence (step/min) | Control (Shoes only): Mean 70.57 (SD: 2.42) Intervention 1 (Silicone AFO): Not reported  Intervention 2 (Codivilla): Mean 71.91 (SD:2.36)  Intervention 3 (Soft foot insert): Mean 76.88 (SD:4.5)  Statistical differences between groups not applicable |
| Right Stance (% gait cycle) | Control (Shoes only): 65.8 Intervention 1 (Silicone AFO): Not reported  Intervention 2 (Codivilla): 61.5  Intervention 3 (Soft foot insert): 62.7  Statistical differences between groups not applicable |
| Left Stance (% gait cycle) | Control (Shoes only): 59.5  Intervention 1 (Silicone AFO): Not reported  Intervention 2 (Codivilla): 60.5  Intervention 3 (Soft foot insert): 61.3  Statistical differences between groups not applicable |
| Right step length (m) | Control (Shoes only): 0.47 Intervention 1 (Silicone AFO): Not reported  Intervention 2 (Codivilla): 0.61  Intervention 3 (Soft foot insert): 0.62  Statistical differences between groups not applicable |
| Left step length (m) | Control (Shoes only): 0.62 Intervention 1 (Silicone AFO): Not reported  Intervention 2 (Codivilla): 0.56  Intervention 3 (Soft foot insert): 0.63  Statistical differences between groups not applicable |
| Right step width (m) | Control (Shoes only): 0.18 Intervention 1 (Silicone AFO): Not reported  Intervention 2 (Codivilla): 0.23  Intervention 3 (Soft foot insert): 0.13  Statistical differences between groups not applicable |
| Left step width (m) | Control (Shoes only): 0.21 Intervention 1 (Silicone AFO): Not reported  Intervention 2 (Codivilla): 0.20  Intervention 3 (Soft foot insert): 0.13  Statistical differences between groups not applicable |
| **Wojciechowski et al. 2022** | |
| Maximum ankle dorsiflexion in swing (˚) | Control (Shoes only) mean -1.1 (SD 8.8) Intervention 1 (Traditional AFO): mean 1.7 (SD 3.7) Intervention 2 (3D printed replica AFO): mean 1.1 (SD 4.1) Intervention 3 (3D printed redesigned AFO): Not reported No significant differences between groups |
| Normalised walking speed | Control (Shoes only) mean 0.4 (SD 0.0) Intervention 1 (Traditional AFO): mean 0.4 (SD 0.1) Intervention 2 (3D printed replica AFO): mean 0.4 (SD 0.1) Intervention 3 (3D printed redesigned AFO): Not reported No significant differences between groups |
| Normalised stride length | Control (Shoes only) mean 1.4 (SD 0.1) Intervention 1 (Traditional AFO): mean 1.5 (SD 0.2) Intervention 2 (3D printed replica AFO): mean 1.5 (SD 0.2) Intervention 3 (3D printed redesigned AFO): Not reported No significant differences between groups |
| Normalised cadence | Control (Shoes only) mean 0.5 (SD 0.0) Intervention 1 (Traditional AFO): mean 0.5 (SD 0.1) Intervention 2 (3D printed replica AFO): mean 0.5 (SD 0.0) Intervention 3 (3D printed redesigned AFO): Not reported No significant differences between groups |
| Maximum ankle dorsiflexion in stance (˚) | Control (Shoes only) mean 16.1 (SD 4.8) Intervention 1 (Traditional AFO): mean 15.7 (SD 3.8) Intervention 2 (3D printed replica AFO): mean 16.1 (SD 3.9) Intervention 3 (3D printed redesigned AFO): Not reported No significant differences between groups |
| Timing of maximum ankle dorsiflexion in stance (% gait cycle) | Control (Shoes only) mean 49.3 (SD 6.0) Intervention 1 (Traditional AFO): mean 51.4 (SD 3.0) Intervention 2 (3D printed replica AFO): mean 50.2 (SD 5.1) Intervention 3 (3D printed redesigned AFO): Not reported No significant differences between groups |
| Maximum ankle plantarflexion at push-off (˚) | Control (Shoes only) mean -15.6 (SD 0.3) Intervention 1 (Traditional AFO): mean -4.3 (SD 2.5) Intervention 2 (3D printed replica AFO): mean -5.1 (SD 5.1) Intervention 3 (3D printed redesigned AFO): Not reported Significant difference (<0.05) compared to shoes |
| Maximum ankle dorsiflexion in the last 1/3 of swing (˚) | Control (Shoes only) mean -1.3 (SD 8.6) Intervention 1 (Traditional AFO): mean 1.7 (SD 3.7) Intervention 2 (3D printed replica AFO): mean 1.1 (SD 4.1) Intervention 3 (3D printed redesigned AFO): Not reported No significant differences between groups |
| Ankle dorsiflexion at initial contact (˚) | Control (Shoes only) mean -5.9 (SD 7.6) Intervention 1 (Traditional AFO): mean 0.0 (SD 3.5) Intervention 2 (3D printed replica AFO): mean -0.6 (SD 3.7) Intervention 3 (3D printed redesigned AFO): Not reported Significant difference (<0.05) compared to shoes |
| Foot progression angle at 25% (˚) | Control (Shoes only) mean -7.8 (SD 16.8) Intervention 1 (Traditional AFO): mean -7.2 (SD 14.9) Intervention 2 (3D printed replica AFO): mean -7.1 (SD 15.0) Intervention 3 (3D printed redesigned AFO): Not reported No significant differences between groups |
| Thigh-foot angle at 25% (˚) | Control (Shoes only) mean -5.1 (SD 16.9) Intervention 1 (Traditional AFO): mean -3.2 (SD 15.9) Intervention 2 (3D printed replica AFO): mean -2.7 (SD 14.6) Intervention 3 (3D printed redesigned AFO): Not reported No significant differences between groups |
| Knee flexion at initial contact (˚) | Control (Shoes only) mean 10.0 (SD 6.5) Intervention 1 (Traditional AFO): mean 7.8 (SD 7.3) Intervention 2 (3D printed replica AFO): mean 7.6 (SD 7.3) Intervention 3 (3D printed redesigned AFO): Not reported No significant differences between groups |
| Maximum knee flexion in loading response (˚) | Control (Shoes only) mean 25.1 (SD 10.1) Intervention 1 (Traditional AFO): mean 19.5 (SD 13.1) Intervention 2 (3D printed replica AFO): mean 19.6 (SD 13.9) Intervention 3 (3D printed redesigned AFO): Not reported No significant differences between groups |
| Minimum knee flexion in stance (˚) | Control (Shoes only) mean 5.7 (SD 12.1) Intervention 1 (Traditional AFO): mean 4.7 (SD 11.0) Intervention 2 (3D printed replica AFO): mean 5.5 (SD 12.0) Intervention 3 (3D printed redesigned AFO): Not reported No significant differences between groups |
| Maximum knee flexion in swing (˚) | Control (Shoes only) mean 74.0 (SD 8.4) Intervention 1 (Traditional AFO): mean 73.2 (SD 6.9) Intervention 2 (3D printed replica AFO): mean 74.2 (SD 7.5) Intervention 3 (3D printed redesigned AFO): Not reported No significant differences between groups |
| Minimum hip flexion (˚) | Control (Shoes only) mean -3.0 (SD 10.5) Intervention 1 (Traditional AFO): mean -1.7 (SD 9.3) Intervention 2 (3D printed replica AFO): mean -2.8 (SD 10.2) Intervention 3 (3D printed redesigned AFO): Not reported No significant differences between groups |
| Maximum hip flexion in swing (˚) | Control (Shoes only) mean 45.1 (SD 10.0) Intervention 1 (Traditional AFO): mean 44.8 (SD 9.6) Intervention 2 (3D printed replica AFO): mean 45.0 (SD 10.5) Intervention 3 (3D printed redesigned AFO): Not reported No significant differences between groups |
| Hip flexion range of motion swing (max in swing – initial contact) (˚) | Control (Shoes only) mean 6.4 (SD 7.0) Intervention 1 (Traditional AFO): mean 4.9 (SD 4.6) Intervention 2 (3D printed replica AFO): mean 5.0 (SD 3.5) Intervention 3 (3D printed redesigned AFO): Not reported No significant differences between groups |
| Maximum hip adduction in stance (˚) | Control (Shoes only) mean 5.1 (SD 3.5) Intervention 1 (Traditional AFO): mean 4.9 (SD 4.6) Intervention 2 (3D printed replica AFO): mean 5.0 (SD 3.5) Intervention 3 (3D printed redesigned AFO): Not reported No significant differences between groups |
| Maximum hip adduction in swing (˚) | Control (Shoes only) mean -0.1 (SD 4.8) Intervention 1 (Traditional AFO): mean 0.6 (SD 3.5) Intervention 2 (3D printed replica AFO): mean -0.2 (SD 4.0) Intervention 3 (3D printed redesigned AFO): Not reported No significant differences between groups |
| Hip rotation at 25% (˚) | Control (Shoes only) mean -7.5 (SD 10.9) Intervention 1 (Traditional AFO): mean -7.6 (SD 10.4) Intervention 2 (3D printed replica AFO): mean -7.3 (SD 9.6) Intervention 3 (3D printed redesigned AFO): Not reported No significant differences between groups |
| Maximum pelvic tilt (˚) | Control (Shoes only) mean 13.9 (SD 6.9) Intervention 1 (Traditional AFO): mean 15.0 (SD 7.4) Intervention 2 (3D printed replica AFO): mean 14.3 (SD 7.2) Intervention 3 (3D printed redesigned AFO): Not reported No significant differences between groups |
| Maximum pelvic obliquity in swing (˚) | Control (Shoes only) mean 3.1 (SD 2.1) Intervention 1 (Traditional AFO): mean 2.7 (SD 2.8) Intervention 2 (3D printed replica AFO): mean 3.0 (SD 2.1) Intervention 3 (3D printed redesigned AFO): Not reported No significant differences between groups |
| Pelvic obliquity ROM in swing (˚) | Control (Shoes only) mean 5.9 (SD 1.9) Intervention 1 (Traditional AFO): mean 5.0 (SD 1.6) Intervention 2 (3D printed replica AFO): mean 5.4 (SD 1.5) Intervention 3 (3D printed redesigned AFO): Not reported No significant differences between groups |
| Pelvic rotation at 25% (˚) | Control (Shoes only) mean 4.8 (3.6) Intervention 1 (Traditional AFO): mean 3.5 (2.9) Intervention 2 (3D printed replica AFO): mean 2.9 (SD 3.30) Intervention 3 (3D printed redesigned AFO): Not reported No significant differences between groups |
| AFO = ankle foot orthosis; ROM = range of motion. | |

| ***Supporting Information 4.*** *Impact of AFOs on gait kinetics* | |
| --- | --- |
| **Outcome** | **Differences between interventions and comparators** |
| **Bean et al. 2001** | |
| Rate pressure product at 22.8 ml of O^2^/kg/min (beats/min·mm Hg) | Control (existing AFO): 25600  Intervention 1 (customised semirigid AFO): 19100  Statistical differences between groups not applicable |
| Rate pressure product at 17.5 ml of O^2^/kg/min (beats/min·mm Hg) | Control (existing AFO): 27100  Intervention 1 (customised semirigid AFO): 21200  Statistical differences between groups not applicable |
| Time to reach 60% peak oxygen consumption (Vo2) (min) | Control (existing AFO): 8.19  Intervention 1 (customised semirigid AFO): 11.06  Statistical differences between groups not applicable |
| Time to reach 75% peak oxygen consumption (Vo2) (min) | Control (existing AFO): 11.06  Intervention 1 (customised semirigid AFO): 16.90  Statistical differences between groups not applicable |
| **Borghi et al. 2023** | |
| Maximum ankle push-off power (W) | Control (barefoot): mean 33.83 (SD 36.41)  Intervention 1 (Silicone AFO): mean 22.25 3.89)  Intervention 2 (Botter AFO): mean 39.00 (SD 11.27)  Statistical differences between groups not reported |
| Maximum pre-swing ankle moment at push-off (Nm) | Control (barefoot): mean 3.50 (SD 3.12)  Intervention 1 (Silicone AFO): mean 5.25 (SD 0.35)  Intervention 2 (Botter AFO): mean 5.17 (SD 2.25)  Statistical differences between groups not reported |
| Peak vertical GRF at push off (% body weight) | Control (barefoot): mean 107.17 (SD 4.80)  Intervention 1 (Silicone AFO): mean 114.50 (SD 6.36)  Intervention 2 (Botter AFO): mean 107.83 (SD 3.40)  Statistical differences between groups not reported |
| Maximum hip power at push off (W) | Control (barefoot): mean 24.17 (SD 9.88)  Intervention 1 (Silicone AFO): mean 34.00 (SD 16.26)  Intervention 2 (Botter AFO): mean 27.00 (SD 5.00)  Statistical differences between groups not reported |
| **Del Bianco & Fatone 2008** | |
| Peak ankle moment in loading response (Nm/kg) (dorsiflexion -/plantarflexion +) | Control (Shoes only): -0.001  Intervention 1 (Posterior leaf spring AFO): -0.248  Intervention 2 (Silicone AFO): -0.00025  Statistical differences between comparisons not applicable |
| Peak ankle plantarflexion moment in stance (Nm/kg) | Control (Shoes only): 0.706  Intervention 1 (Posterior leaf spring AFO): 0.992  Intervention 2 (Silicone AFO): 0.8275  Statistical differences between comparisons not applicable |
| Moment corresponding to minimum knee angle in stance (Nm/kg) (flexion -/extension+) | Control (Shoes only): 0.0675  Intervention 1 (Posterior leaf spring AFO): -0.213  Intervention 2 (Silicone AFO): -0.254  Statistical differences between comparisons not applicable |
| Heel strike transient (% body weight) | Control (Shoes only): 0.132  Intervention 1 (Posterior leaf spring AFO): Not reported  Intervention 2 (Silicone AFO): 0.255  Statistical differences between comparisons not applicable |
| First peak of vertical GRF (% body weight) | Control (Shoes only): mean 1.035  Intervention 1 (Posterior leaf spring AFO): 1.16  Intervention 2 (Silicone AFO): 1.09  Statistical differences between comparisons not applicable |
| **Menotti et al. 2014** | |
| Walking energy cost per unit of time (WECt) slow (calculated as the amount of oxygen uptake per unit of body mass and per unit of time) (J/kg/min) | Control (Shoes only): mean 136.10 (SE 7.86)  Intervention 1 (Anterior elastic AFO): mean 115.31 (SD 6.08)  No significant differences between conditions |
| Walking energy cost per unit of time (WECt) comfortable (calculated as the amount of oxygen uptake per unit of body mass and per unit of time) (J/kg/min) | Control (Shoes only): mean 159.41 (SE 5.58)  Intervention 1 (Anterior elastic AFO): mean 143.95 (SD 10.64)  Significantly lower in control (P<0.05) |
| Walking energy cost per unit of time (WECt) fast (calculated as the amount of oxygen uptake per unit of body mass and per unit of time) (J/kg/min) | Control (Shoes only): mean 248.88 (SE 18.50)  Intervention 1 (Anterior elastic AFO): mean 268.65 (SD 26.86)  Significantly higher in control (P<0.05) |
| Walking energy cost per unit of distance slow (WECd) (calculated as the amount of oxygen uptake per unit of body mass and per unit of distance) (J/kg/m) | Control (Shoes only): mean 2.68 (SE 0.11)  Intervention 1 (Anterior elastic AFO): mean 2.49 (SD 0.15)  No significant differences between conditions |
| Walking energy cost per unit of distance comfortable (WECd) (calculated as the amount of oxygen uptake per unit of body mass and per unit of distance) (J/kg/m) | Control (Shoes only): mean 2.73 (SE 0.14)  Intervention 1 (Anterior elastic AFO): mean 2.43 (SD 0.17)  Significantly lower in control (P<0.05) |
| Walking energy cost per unit of distance fast (WECd) (calculated as the amount of oxygen uptake per unit of body mass and per unit of distance) (J/kg/m) | Control (Shoes only): mean 3.17 (SE 0.13)  Intervention 1 (Anterior elastic AFO): mean 3.35 (SD 0.25)  Significantly higher in control (P<0.05) |
| **Ounpuu et al. 2021** | |
| Peak ankle plantarflexor moment (Nm/kg) | Control (Barefoot): mean 0.71 (SD 0.30)  Intervention 1 (AFO): mean 0.85 (SD 0.29)  Significantly higher in AFO (P=0.001) |
| Peak ankle plantarflexor power generation (W/kg) | Control (Barefoot): mean 1.63 (SD 1.24)  Intervention 1 (AFO): mean 1.25 (SD 0.55)  No significant difference between comparisons. |
| Peak ankle dorsiflexor moment loading (Nm/kg) | Control (Barefoot): mean 0.02 (SD 0.04)  Intervention 1 (AFO): mean -0.19 (SD 0.10)  Significantly lower in AFO (p= 0.0001) |
| Peak hip power generation at toe off (W/kg) | Control (Barefoot): mean 1.35 (SD 0.79)  Intervention 1 (AFO): mean 1.20 (SD 0.71)  No significant difference between comparisons. |
| **Van der Wilk et al. 2018** | |
| Powered plantarflexion maximum moment (Nm) | Control (existing AFO): 61  Intervention 1 (ADJUST AFO stiff): 54 Intervention 2 (ADJUST AFO stiff/flexible): 54  Intervention 3 (ADJUST AFO flexible): 61  Intervention 4 (ADJUST AFO flexible/stiff): 61  Statistical differences between groups not reported |
| Powered plantarflexion maximum power (W) | Control (existing AFO): 84  Intervention 1 (ADJUST AFO stiff): 54 Intervention 2 (ADJUST AFO stiff/flexible): 54  Intervention 3 (ADJUST AFO flexible): 61  Intervention 4 (ADJUST AFO flexible/stiff): 61  Statistical differences between groups not reported |
| **Wojciechowski et al. 2022** | |
| Maximum ankle dorsiflexor moment in loading response (Nm/kg) | Control (Shoes only): mean -0.1 (SD 0.1) Intervention 1(Traditional AFO): mean -0.3 (SD 0.1) Intervention 2 (3D printed replica AFO): mean -0.3 (SD 0.1) Intervention 3 (3D printed redesigned AFO): Not reported Significant difference compared to shoes (P<0.001) |
| Maximum ankle plantarflexor moment (Nm/kg) | Control (Shoes only): mean 0.9 (SD 0.3) Intervention 1 (Traditional AFO): mean 1.1 (SD 0.3) Intervention 2 (3D printed replica AFO): mean 1.1 (SD 0.3) Intervention 3 (3D printed redesigned AFO): Not reported No significant differences between comparisons |
| Maximum ankle power generation in mid-stance (W/kg) | Control (Shoes only): mean 0.0 (SD 0.1) Intervention 1 (Traditional AFO): mean 0.0 (SD 0.1) Intervention 2 (3D printed replica AFO): mean 0.1 (SD 0.2) Intervention 3 (3D printed redesigned AFOs): Not reported Significant difference compared to shoes (P<0.05) |
| Maximum ankle power at push-off (W/kg) | Control (Shoes only): mean 1.8 (SD 0.8) Intervention 1 (Traditional AFO): mean 1.6 (SD 0.8) Intervention 2 (3D printed replica AFO): mean 1.6 (SD 0.7) Intervention 3 (3D printed redesigned AFO): Not reported No significant differences between comparisons |
| Maximum knee flexor moment in single support (Nm/kg) | Control (Shoes only): mean -0.1 (SD 0.2) Intervention 1 (Traditional AFO): mean -0.2 (SD 0.2) Intervention 2 (3D printed replica AFO): mean -0.2 (SD 0.2) Intervention 3 (3D printed redesigned AFOs: Not reported No significant differences between comparisons |
| Maximum extensor moment in stance (Nm/kg) | Control (Shoes only): mean 0.6 (SD 0.3) Intervention 1 (Traditional AFO): mean 0.5 (SD 0.3) Intervention 2 (3D printed replica AFO): mean 0.5 (SD 0.3) Intervention 3 (3D printed redesigned AFO): Not reported No significant differences between comparisons |
| Mean sagittal plane knee moment in stance (Nm/kg) | Control (Shoes only): mean 0.1 (SD 0.2) Intervention 1 (Traditional AFOs: mean 0.1 (SD 0.1) Intervention 2 (3D printed replica AFO): mean 0.1 (SD 0.1) Intervention 3 (3D printed redesigned AFO): Not reported No significant differences between comparisons |
| Maximum hip abductor moment in terminal stance (Nm/kg) | Control (Shoes only): mean 0.3 (SD 0.1) Intervention 1 (Traditional AFO): mean 0.3 (SD 0.1) Intervention 2 (3D printed replica AFO): mean 0.3 (SD 0.1) Intervention 3 (3D printed redesigned AFO): Not reported No significant differences between comparisons |
| AFO = ankle-foot orthosis; 3D = three dimensional; GRF = ground reaction force. | |

| ***Supporting Information 5.*** *Impact of AFOs on postural stability/balance (n = 4 studies)* | |
| --- | --- |
| **Outcome** | **Differences between interventions and comparators** |
| **Borghi et al. 2023** | |
| Mean CoP velocity (mm/s) | Control (Barefoot): mean 35.0 SD (21.2)  Intervention 1 (Silicone AFO): mean 19.5 (SD 0.7)  Intervention 2 (Botter AFO): mean 20.7 (SD 2.5)  Statistical differences between groups not reported |
| Root mean square of CoP displacement (mm) | Control (Barefoot): mean 11.5 (SD 3.5)  Intervention 1 (Silicone AFO): mean 7.0 (SD 1.4)  Intervention 2 (Botter AFO): mean 10.0 (SD 3.5)  Statistical differences between groups not reported |
| **Burke et al. 2021** | |
| Time held (s) with eyes open on firm surface | Control (Shoes only): mean 28.9 Intervention 1 (Carbon ground reaction AFO): mean 30.0  No significant difference between conditions |
| Sway score with eyes open on firm surface (scored from 1 minimal sway to 4 loss of balance/fall) | Control (Shoes only): Range 1-2 Intervention 1 (Carbon ground reaction AFO): Range 1-1  No significant difference between conditions |
| Time held (s) with eyes closed on firm surface | Control (Shoes only): Mean 24.0 Intervention 1 (Carbon ground reaction AFO): Mean 27.0  No significant difference between conditions |
| Sway score with eyes closed on firm surface (scored from 1 minimal sway to 4 loss of balance/fall) | Control (Shoes only): Range 1.5-4 Intervention 1 (Carbon ground reaction AFO): Range 1-4  Statistical differences between comparison no significant difference between conditions |
| Time held (s) with eyes open on foam surface | Control (Shoes only): Mean 28.2 Intervention 1 (Carbon ground reaction AFO): Mean 28.3  No significant difference between conditions |
| Sway score with eyes open on foam surface (scored from 1 minimal sway to 4 loss of balance/fall) | Control (Shoes only): Range 1-3.5 Intervention 1 (Carbon ground reaction AFO): Range 1-4  No significant difference between conditions |
| Time held (s) with eyes closed on foam surface | Control (Shoes only): Mean 7.7 Intervention 1 (Carbon ground reaction AFO): Mean 10.2  No significant difference between conditions |
| Sway score with eyes closed on foam surface (scored from 1 minimal sway to 4 loss of balance/fall) | Control (Shoes only): Range 2-4 Intervention 1 (Carbon ground reaction AFO): Range 3-4  No significant difference between conditions |
| Time held (s) with eyes open on firm surface | Control (Shoes only): mean 28.9 Intervention 1 (Carbon ground reaction AFO): mean 30.0  No significant difference between conditions |
| **Guillebastre et al. 2011** | |
| Centre of Pressure trajectory area (mm2) | Control (Shoes with/ without brace): mean 318.72 (SD 213.35)  Intervention 1 (Plastic AFO): mean 232.51 (SD 148.04)  Intervention 2 (Elastic AFO): mean 263.86 (SD 228.16)  Significantly greater in control vs Intervention 1 (P<0.05) |
| Centre of pressure anterior posterior displacement amplitude (mm) | Control (Shoes with/ without brace): mean 0.76 (SD 0.17)  Intervention 1 (Plastic AFO): mean 0.69 (SD 0.18)  Intervention 2 (Elastic AFO): mean 0.71 (SD 0.27)  No significant differences between comparisons. |
| - Centra of pressure medial lateral displacement amplitude (mm) | Control (Shoes with/ without brace): mean 0.45 (SD 0.25)  Intervention 1 (Plastic AFO): mean 0.37 (SD 0.18)  Intervention 2 (Elastic AFO): mean 0.39 (0.20)  No significant differences between comparisons. |
| - Centre of Pressure anterior posterior displacement frequency (Hz) | Control (Shoes with/ without brace): mean 0.42 (SD 0.15)  Intervention 1 (Plastic AFO): mean 0.45 (SD 0.13)  Intervention 2 (Elastic AFO): mean 0.43 (SD 0.15)  No significant differences between comparisons. |
| - Centre of Pressure medial lateral displacement frequency (Hz) | Control (Shoes with/ without brace): mean 0.45 (SD 0.11)  Intervention 1 (Plastic AFO): mean 0.46 (SD 0.11)  Intervention 2 (Elastic AFO): mean 0.45 (SD 0.12)  No significant differences between comparisons. |
| **Pereira et al. 2014** | |
| Tinetti scale (assesses balance and gait with total scores ranging from 0 to 28) | Control (Shoes only): mean 22 (SD 4)  Intervention 1 (AFO): mean 23 (SD 3)  No significant difference between comparisons |
| AFO = ankle foot orthosis; CoP = centre of pressure. | |
